# Supplementary material for: A multifunctional smart field-programmable radio frequency surface
Source: Nat Commun. 2024 May 13;15:4042. doi: 10.1038/s41467-024-48242-z (PMC11091106; doi:10.1038/s41467-024-48242-z)
Supplement: Supplementary file 1 — Supplementary Information [file 41467_2024_48242_MOESM1_ESM.pdf]

A Multifunctional Smart Field-Programmable Radio Frequency Surface

This supplementary information contains the following sections:

Supplementary Note 1: FPRFS structure and digital programming scheme

Supplementary Note 2: Current dipole image theory

Supplementary Note 3: Current dipole modeling and calculation details

Supplementary Note 4: Rectangular segment width calculation

Supplementary Note 5: Radian electrical length analysis for the IMN

Supplementary Note 6: Impedance matching capability calculation

Supplementary Note 7: Impedance matching bandwidth

Supplementary Note 8: FPRFS-based antenna's polarization programmability

Supplementary Note 9: PIN diode modeling and characterization

Supplementary Note 10: The cruciform RF switching unit

Supplementary Note 11: Comparative analysis of antenna efficiency

Supplementary Note 12: Reconfigurable antenna efficiency comparison

Supplementary Note 13: Integrating Advanced Optimization Algorithms

Supplementary Note 14: Direction coupler modeling and characterization

Supplementary Note 15: Experimental setup discussion

Supplementary Note 16: Integrating multi-objective optimization methods

Supplementary Note 17: FPRFS control and visualization software interface

Supplementary References

Supplementary Note 1: FPRFS structure and digital programming scheme

The Field-Programmable Radio Frequency Surface (FPRFS) structure is shown in Supplementary Fig. 1 and compared with the structure of a Field-Programmable Gate Array (FPGA). They share

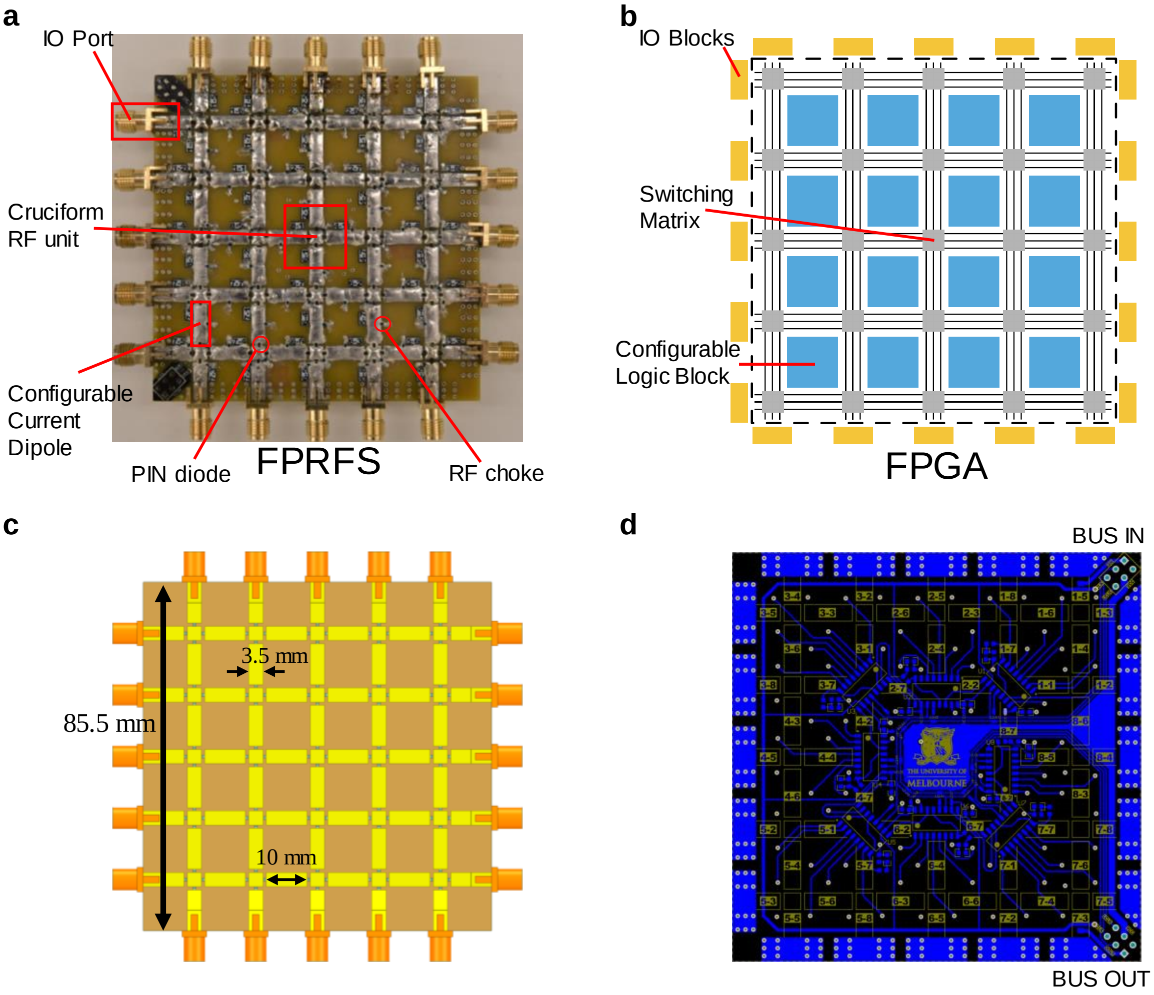

Supplementary Fig. 1 | Structures of the FPRFS and FPGA. a Image of the fabricated FPRFS. **b** Schematic of an FPGA. **c** Image of the FPRFS simulation model in HFSS. **d** The bottom side of the FPRFS with digital programming circuits.

similarities including configurable IO ports, signal routing capability, and digital programmability. The major difference is the basic configurable block. For FPGAs, the logic block is the basic unit for constructing digital circuits. While for FPRFSs, the current dipole is the basic unit for emulating complicated current distribution patterns. The HFSS simulation model created for the FPRFS is shown in Supplementary Fig. 1c. The basic rectangular segment pixel dimension is chosen to be 10 mm × 3.5 mm and is justified in the paper's main text. The mid-square conductor size is 3.5 mm × 3.5 mm. A mid square with four adjacent rectangular segments forms a cruciform RF switching unit. A major limitation for scaling down the segment pixel dimension further is from the PIN diode-based RF switches applied, with an equivalent electrical length of about 5 mm in the GHz range. Further reducing the segment pixel dimension will lead to PIN diode-based RF switch dominating the reconfigurable current path in the FPRFS, causing unpredictable behaviors and inefficient radiation through the dipole conductor segments. An array of 5 × 5 cruciform RF switching units consisting of 60 segment pixels forms the FPRFS in this work. The overall dimension of the FPRFS is 85.5 mm × 85.5 mm. The weight of a single FPRFS (without SMA connectors) is about 30 grams contributed mostly by the PCB substrate. Along the FPRFS edge, there are 20 IO ports. In this work, not all SMA connectors are mounted for experimental convenience. When programmed as impedance matching networks (IMN), the loaded branch stub terminals do not have mounted SMA connectors. The bottom side PCB design with onboard memory and biasing circuits is shown in Supplementary Fig. 1d, with bitstream programming bus input and output for multi-FPRFS cascading extendibility. The FPRFS is designed and fabricated with such a dimension to cater to RF applications in the UHF range covering the commonly used 2.4-2.5 GHz. More importantly, the FPRFS proposed in this work is a concept with scalability. Given RF switches with shorter equivalent electrical lengths and higher integration levels, further

reducing the pixel size is conceivable for a more compact design and enhanced pattern programming resolution, catering to practical handheld device applications.

The digital programming scheme is shown in Supplementary Fig. 2. The speed of the local microcontroller programming the FPRFS is fast and the bitstream waveform for programming a single-FPRFS as a 2×2-2-1 patch antenna is shown in Supplementary Fig. 2b, which takes about 25 us. The maximum programming frequency of 40,000 Hz can be achieved. This time is proportional to the number of boards under configuration and the bitstream waveform for programming a double-FPRFS topology is shown in Supplementary Fig. 2c, which takes 50 us. As the FPRFS resolution increases, both the pattern versatility and programming time increase.

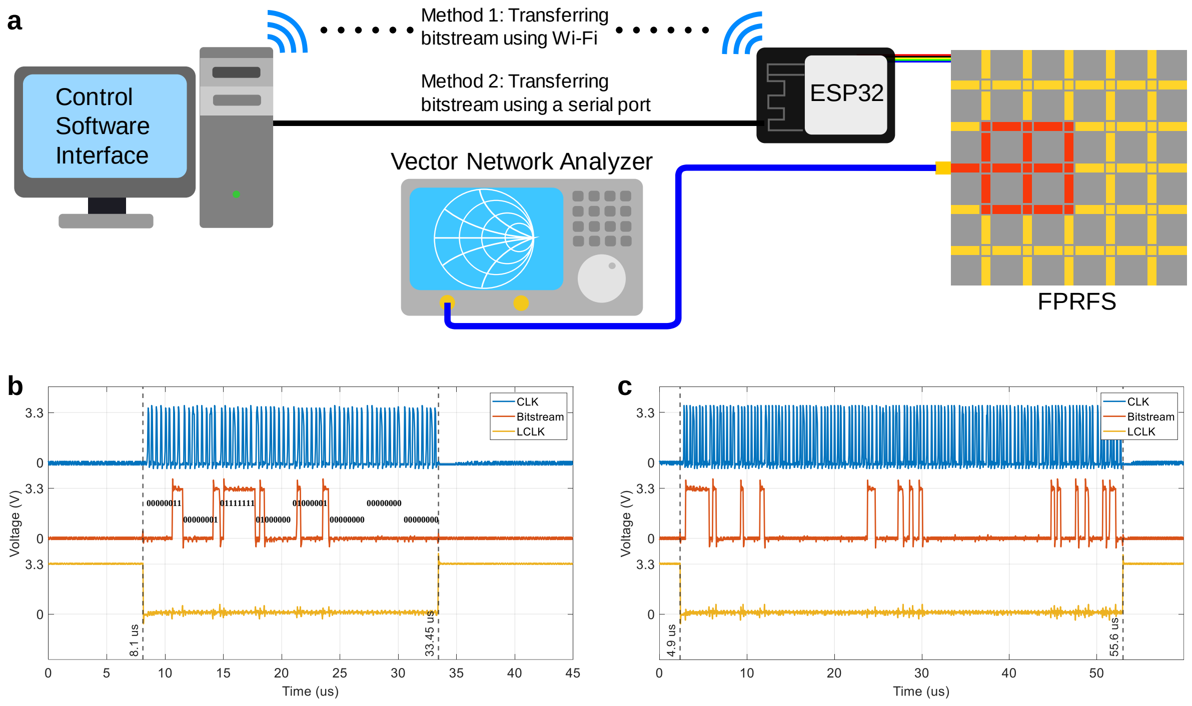

Supplementary Fig. 2 | FPRFS digital programming scheme. a Digital programming scheme of the FPRFS. **b** Measured bitstream waveform for programming a single-FPRFS structure. **c** Measured bitstream waveform for programming a cascaded double-FPRFS structure.

Supplementary Note 2: Current dipole image theory

Considering the beneath-ground plane as an infinite PEC, as per the image theory[1], the field distribution of a source in space is the superposition of those for itself and its virtual imaged source across the PEC plane. As the PEC plane is parallel to the loop antenna plane and the source is an electric dipole in this case, its virtual imaged source is under the PEC at the same distance with the same magnitude but reversed vector direction, as shown in Supplementary Fig. 3a. The distances from the observation point to the center of the electric dipole itself, its virtual image, and the intersection point of their line and the PEC plane are defined as r_{up}, r_{below}, and r_{mid}, as shown in Supplementary Fig. 3b. The angle between the distance r_{mid} and the z-axis is defined as θ_{mid}. Therefore using the law of cosine:

$$r_{\mathrm{up}} = \sqrt{r_{\mathrm{mid}}^{2} + h^{2} - 2r_{\mathrm{mid}} h \cos\theta_{\mathrm{mid}}}\ ,\ r_{\mathrm{below}} = \sqrt{r_{\mathrm{mid}}^{2} + h^{2} + 2r_{\mathrm{mid}} h \cos(\pi - \theta_{\mathrm{mid}})}\ . \quad (1)$$

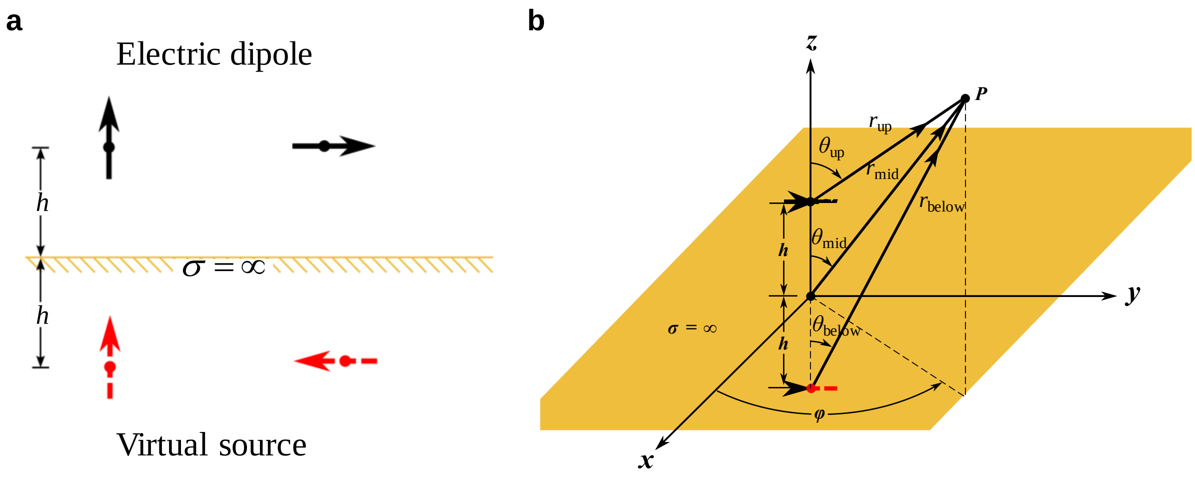

Supplementary Fig. 3 | Current image theory illustration. a Vertical and horizontal electric dipoles above a PEC and their virtual sources. **b** The superpositioned fields of two dipole sources at an observation point P.

Using the binomial expansion and omitting the higher-order terms, their simplified linear expressions are:

$$r_{\text{up}} = r_{\text{mid}} - h\cos\theta_{\text{mid}}\,,\ r_{\text{below}} = r_{\text{below}} + h\cos\theta_{\text{mid}}\,. \tag{2}$$

At far-field, the magnitude variations are not critical and the phase variations are equal to:

$$e^{-jkr_{\text{up}}} - e^{-jkr_{\text{below}}} = e^{-jk(r - h\cos\theta_{\text{mid}})} - e^{-jk(r + h\cos\theta_{\text{mid}})} = e^{-jkr}\cdot\ 2j\sin(kh\cos\theta_{\text{mid}})\,. \tag{3}$$

θ_{mid} is approximated to be θ thus the array factor of $2j\sin(kh\cos\theta_{\text{mid}})$ is derived for calculating the total E-field and H-field distributions.

Supplementary Note 3: Current dipole modeling and calculation details

A Cartesian coordinate system is created as shown, and the dipoles' vector potential functions can be expressed by

$$\mathbf{A}_n(x,y,z) = (-1)^n\cdot\ \mu I_0 l_0\cdot\ e^{-jkr_n}/8\pi r_n\cdot\ \hat{\mathbf{a}}_y\,,\ n = 0,1,2,3,4\,,$$

$$\mathbf{A}_n(x,y,z) = (-1)^n\cdot\ \mu I_0 l_0\cdot\ e^{-jkr_n}/8\pi r_n\cdot\ \hat{\mathbf{a}}_x\,,\ n = 5,6,7,8\,, \tag{4}$$

where I_0 is the uniform phase current magnitude in the loop antenna. All calculations are based on the far-field approximation, where $r \gg l_0$. The H field vector for each dipole can be derived as for dipole 0:

$$\mathbf{H}_0 = \frac{1}{\mu}\nabla\times\mathbf{A}_0 \simeq jkI_0 l_0 \cdot e^{-jkr} / 8\pi r^2 \cdot (z\cdot\hat{\mathbf{a}}_x - x\cdot\hat{\mathbf{a}}_z)\,.$$

(5)

Let $K_n = jkI_0 l_0 \cdot\ e^{-jkr_n} / 8\pi r_n{}^2$ and calculate the total H field, which is a superposition of H field vectors of nine small dipoles. This term K_n existing in the H-field expression of each small dipole n contains the distance r_n from the observation point to the center of each small dipole n.

For the 9-segment loop antenna model and the established Cartesian coordinate system, all r_n can be expressed in Cartesian coordinates and polar coordinates as:

$$\begin{cases} r_0 = \sqrt{x^2+y^2+z^2} = r \\ r_1 = \sqrt{(x-l_0)^2+(y-l_0)^2+z^2} = \sqrt{r^2+2l^2-2lr\sin\theta(\sin\phi+\cos\phi)} \\ r_2 = \sqrt{x^2+(y-l_0)^2+z^2} = \sqrt{r^2+l^2-2lr\sin\theta\sin\phi} \\ r_3 = \sqrt{(x-l_0)^2+(y+l_0)^2+z^2} = \sqrt{r^2+2l^2+2lr\sin\theta(\sin\phi-\cos\phi)} \\ r_4 = \sqrt{x^2+(y+l_0)^2+z^2} = \sqrt{r^2+l^2+2lr\sin\theta\sin\phi} \\ r_5 = \sqrt{(x-\frac{3}{2}l_0)^2+(y+\frac{1}{2}l_0)^2+z^2} = \sqrt{r^2+\frac{5}{2}l^2+lr\sin\theta(\sin\phi-3\cos\phi)} \\ r_6 = \sqrt{(x-\frac{3}{2}l_0)^2+(y-\frac{1}{2}l_0)^2+z^2} = \sqrt{r^2+\frac{5}{2}l^2-lr\sin\theta(\sin\phi+3\cos\phi)} \\ r_7 = \sqrt{(x-\frac{1}{2}l_0)^2+(y+\frac{3}{2}l_0)^2+z^2} = \sqrt{r^2+\frac{5}{2}l^2+lr\sin\theta(3\sin\phi-\cos\phi)} \\ r_8 = \sqrt{(x-\frac{1}{2}l_0)^2+(y-\frac{3}{2}l_0)^2+z^2} = \sqrt{r^2+\frac{5}{2}l^2-lr\sin\theta(3\sin\phi+\cos\phi)} \end{cases}.$$

(6)

They cannot be assumed to be equal in the far-field because the phase difference e^{-jkr_n} term in K_n is not negligible. Therefore, Maclarurin series expansion is used to approximate the r_n by omitting higher-order terms as:

$$\begin{cases} K_0 = \dfrac{jkI_0 l_0 \cdot \ e^{-jkr}}{8\pi r^2} \\ K_1 = \dfrac{kI_0 l_0 e^{-jkr}}{8\pi r^2}(j - kl_0 \sin\theta(\sin\phi + \cos\phi)) \\ K_2 = \dfrac{kI_0 l_0 e^{-jkr}}{8\pi r^2}(j - kl_0 \sin\theta \sin\phi) \\ K_3 = \dfrac{kI_0 l_0 e^{-jkr}}{8\pi r^2}(j + kl_0 \sin\theta(\sin\phi - \cos\phi)) \\ K_4 = \dfrac{kI_0 l_0 e^{-jkr}}{8\pi r^2}(j + kl_0 \sin\theta \sin\phi) \\ K_5 = \dfrac{kI_0 l_0 e^{-jkr}}{8\pi r^2}(j + \dfrac{1}{2}kl_0 \sin\theta(\sin\phi - 3\cos\phi)) \\ K_6 = \dfrac{kI_0 l_0 e^{-jkr}}{8\pi r^2}(j - \dfrac{1}{2}kl_0 \sin\theta(\sin\phi + 3\cos\phi)) \\ K_7 = \dfrac{kI_0 l_0 e^{-jkr}}{8\pi r^2}(j + \dfrac{1}{2}kl_0 \sin\theta(3\sin\phi - \cos\phi)) \\ K_8 = \dfrac{kI_0 l_0 e^{-jkr}}{8\pi r^2}(j - \dfrac{1}{2}kl_0 \sin\theta(3\sin\phi + \cos\phi)) \end{cases}.$$

(7)

The summed H-field thus can be derived as follows:

$$\begin{aligned} \sum_{n=0}^{8}\mathbf{H}_n &= (K_0 - K_1 + K_2 - K_3 + K_4)\cdot z \cdot \hat{\mathbf{a}}_x + (K_5 - K_6 + K_7 - K_8)\cdot z \cdot \hat{\mathbf{a}}_y \\ &+[-K_0 x + K_1(x - l_0) - K_2 x + K_3(x - l_0) - K_4 x - K_5(y + l_0/2) + K_6(y - l_0/2) \\ &-K_7(y + 3l_0/2) + K_8(y - 3l_0/2)]\cdot \hat{\mathbf{a}}_z \end{aligned}.$$

(8)

Plugging the K_n into Supplementary Equation (8). The total H field is equal to:

$$\begin{cases} H_x = kI_0 l_0 \cdot \ e^{-jkr}/8\pi r^2 \cdot \ (2kl_0 \cdot \ xz/r + j\cdot \ z) \\ H_y = kI_0 l_0 \cdot \ e^{-jkr}/2\pi r^2 \cdot \ (kl_0 \cdot \ yz/r) \\ H_z = -kI_0 l_0 \cdot \ e^{-jkr}/8\pi r^2 \cdot \ (2kl_0 \cdot \ x^2/r + 4kl_0 \cdot \ y^2/r + 6j\cdot \ x) \end{cases}.$$

(9)

The E field $\mathbf{E}=\frac{1}{j\omega\varepsilon}\nabla\times\mathbf{H}$ is equal to:

$$\begin{cases} E_x = k^2 I_0 l_0 \cdot\ e^{-jkr}/2\pi\omega\varepsilon r^3 \cdot\ (kl_0 \cdot\ x^2 y/2r + kl_0 \cdot\ y^3/r + kl_0 \cdot\ yz^2/r + j \cdot\ xy/4) \\ E_y = -k^2 I_0 l_0 \cdot\ e^{-jkr}/2\pi\omega\varepsilon r^3 \cdot\ (kl_0 \cdot\ xz^2/2r + kl_0 \cdot\ x^3/2r + kl_0 \cdot\ xy^2/r + j \cdot\ x^2/4 + j \cdot\ z^2/4) \\ E_z = -k^2 I_0 l_0 \cdot\ e^{-jkr}/2\pi\omega\varepsilon r^3 \cdot\ (kl_0 \cdot\ xyz/2r - j \cdot\ yz/4) \end{cases}.$$

(10)

Considering the infinitely large PEC, the final E and H fields will multiply the array factor $2j\sin(kh\cos\theta)$, and the average Poynting vector is equal to $\mathbf{W}_{\mathrm{av}}=\frac{1}{2}\mathrm{Re}[\mathbf{E}_{\mathrm{total}}\times\mathbf{H}^{*}_{\mathrm{total}}]$ where the resultant component in each of the cartesian coordinates is equal to:

$$\begin{cases} W_x = k^5 I_0^2 l_0^{\,4} \cdot\ \sin^2(kh\cos(\theta))/8\pi^2\omega\varepsilon r^5 \cdot\ x[x^2(x^2+z^2)/r^2 + 4y^2 + (x^2+z^2)/4k^2 l_0^{\,2}] \\ W_y = k^5 I_0^2 l_0^{\,4} \cdot\ \sin^2(kh\cos(\theta))/8\pi^2\omega\varepsilon r^5 \cdot\ y[x^2(x^2+z^2)/r^2 + 4y^2 + (x^2+z^2)/4k^2 l_0^{\,2}] \\ W_z = k^5 I_0^2 l_0^{\,4} \cdot\ \sin^2(kh\cos(\theta))/8\pi^2\omega\varepsilon r^5 \cdot\ z[x^2(x^2+z^2)/r^2 + 4y^2 + (x^2+z^2)/4k^2 l_0^{\,2}] \end{cases},\ z\geq 0.$$

(11)

Transforming these equations to spherical coordinates, we find that:

$$\begin{cases} W_r = k^3 I_0^2 l_0^{\,2} \cdot\ \sin^2(kh\cos(\theta))/32\pi^2\omega\varepsilon r^2 \cdot\ [\cos^4\theta + (1+4k^2 l_0^{\,2})\cos^4\phi\sin^4\theta + \\ \qquad \frac{1}{2}\cos^2\theta(3+4k^2 l_0^{\,2}+(1+4k^2 l_0^{\,2})\cos 2\phi)\sin^2\theta + 16k^2 l_0^{\,2}\sin^2\theta\sin^2\phi + \sin^4\theta\sin^2\phi\cos^2\phi] \\ W_\theta = 0 \\ W_\phi = 0 \end{cases},$$

$$z\geq 0.$$

(12)

The radiation intensity U at $z\geq 0$ is equal to:

$$
\begin{aligned}
U &= r^2 W_{\mathrm{av}} \\
&= k^3 I_0^2 l_0{}^2 \cdot \ \sin^2(kh\cos(\theta)) / 32\pi^2\omega\varepsilon \cdot \ [\cos^4\theta + (1+4k^2 l_0{}^2)\cos^4\phi\sin^4\theta + \\
&\frac{1}{2}\cos^2\theta(3+4k^2 l_0{}^2 + (1+4k^2 l_0{}^2)\cos 2\phi)\sin^2\theta + 16k^2 l_0{}^2\sin^2\theta\sin^2\phi + \sin^4\theta\sin^2\phi\cos^2\phi]
\end{aligned}
\quad . \tag{13}
$$

Substituting the values for θ, and ϕ at a frequency of 2.45 GHz, the calculated radiation patterns in the $\phi = 0°$ and $\phi = 90°$ planes are derived.

Similarly, for the first 11-segment loop antenna model:

$$
\begin{cases}
r_0 = \sqrt{(x+l_0)^2 + y^2 + z^2} = \sqrt{r^2 + l^2 + 2lr\sin\theta\cos\phi} \\
r_1 = \sqrt{(x-l_0)^2 + (y-l_0)^2 + z^2} = \sqrt{r^2 + 2l^2 - 2lr\sin\theta(\sin\phi + \cos\phi)} \\
r_2 = \sqrt{(x+l_0)^2 + (y-l_0)^2 + z^2} = \sqrt{r^2 + 2l^2 - 2lr\sin\theta(\sin\phi - \cos\phi)} \\
r_3 = \sqrt{(x-l_0)^2 + (y+l_0)^2 + z^2} = \sqrt{r^2 + 2l^2 + 2lr\sin\theta(\sin\phi - \cos\phi)} \\
r_4 = \sqrt{(x+l_0)^2 + (y+l_0)^2 + z^2} = \sqrt{r^2 + 2l^2 + 2lr\sin\theta(\sin\phi + \cos\phi)} \\
r_5 = \sqrt{(x-\frac{3}{2}l_0)^2 + (y+\frac{1}{2}l_0)^2 + z^2} = \sqrt{r^2 + \frac{5}{2}l^2 + lr\sin\theta(\sin\phi - 3\cos\phi)} \\
r_6 = \sqrt{(x-\frac{3}{2}l_0)^2 + (y-\frac{1}{2}l_0)^2 + z^2} = \sqrt{r^2 + \frac{5}{2}l^2 - lr\sin\theta(\sin\phi + 3\cos\phi)} \\
r_7 = \sqrt{(x-\frac{1}{2}l_0)^2 + (y+\frac{3}{2}l_0)^2 + z^2} = \sqrt{r^2 + \frac{5}{2}l^2 + lr\sin\theta(3\sin\phi - \cos\phi)} \\
r_8 = \sqrt{(x-\frac{1}{2}l_0)^2 + (y-\frac{3}{2}l_0)^2 + z^2} = \sqrt{r^2 + \frac{5}{2}l^2 - lr\sin\theta(3\sin\phi + \cos\phi)} \\
r_9 = \sqrt{(x+\frac{1}{2}l_0)^2 + (y+\frac{3}{2}l_0)^2 + z^2} = \sqrt{r^2 + \frac{5}{2}l^2 + lr\sin\theta(3\sin\phi + \cos\phi)} \\
r_{10} = \sqrt{(x+\frac{1}{2}l_0)^2 + (y-\frac{3}{2}l_0)^2 + z^2} = \sqrt{r^2 + \frac{5}{2}l^2 - lr\sin\theta(3\sin\phi - \cos\phi)}
\end{cases}
, \tag{14}
$$

$$\begin{cases} K_0 = \dfrac{jkI_0 l_0 \cdot \ e^{-jkr}}{8\pi r^2}(j + kl_0 \sin\theta\cos\phi) \\ K_1 = \dfrac{kI_0 l_0 e^{-jkr}}{8\pi r^2}(j - kl_0 \sin\theta(\sin\phi + \cos\phi)) \\ K_2 = \dfrac{kI_0 l_0 e^{-jkr}}{8\pi r^2}(j - kl_0 \sin\theta(\sin\phi - \cos\phi)) \\ K_3 = \dfrac{kI_0 l_0 e^{-jkr}}{8\pi r^2}(j + kl_0 \sin\theta(\sin\phi - \cos\phi)) \\ K_4 = \dfrac{kI_0 l_0 e^{-jkr}}{8\pi r^2}(j + kl_0 \sin\theta(\sin\phi + \cos\phi)) \\ K_5 = \dfrac{kI_0 l_0 e^{-jkr}}{8\pi r^2}(j + \dfrac{1}{2}kl_0 \sin\theta(\sin\phi - 3\cos\phi)) \\ K_6 = \dfrac{kI_0 l_0 e^{-jkr}}{8\pi r^2}(j - \dfrac{1}{2}kl_0 \sin\theta(\sin\phi + 3\cos\phi)) \\ K_7 = \dfrac{kI_0 l_0 e^{-jkr}}{8\pi r^2}(j + \dfrac{1}{2}kl_0 \sin\theta(3\sin\phi - \cos\phi)) \\ K_8 = \dfrac{kI_0 l_0 e^{-jkr}}{8\pi r^2}(j - \dfrac{1}{2}kl_0 \sin\theta(3\sin\phi + \cos\phi)) \\ K_9 = \dfrac{kI_0 l_0 e^{-jkr}}{8\pi r^2}(j + \dfrac{1}{2}kl_0 \sin\theta(3\sin\phi + \cos\phi)) \\ K_{10} = \dfrac{kI_0 l_0 e^{-jkr}}{8\pi r^2}(j - \dfrac{1}{2}kl_0 \sin\theta(3\sin\phi - \cos\phi)) \end{cases},$$

(15)

$$\begin{aligned} \sum_{n=0}^{10}\mathbf{H}_n &= (K_0 - K_1 + K_2 - K_3 + K_4)\cdot z \cdot \hat{\mathbf{a}}_x + (K_5 - K_6 + K_7 - K_8 + K_9 - K_{10})\cdot z \cdot \hat{\mathbf{a}}_y \\ &+[-K_0(x+l_0) + K_1(x-l_0) - K_2(x+l_0) + K_3(x-l_0) - K_4(x+l_0) - K_5(y+l_0/2) \\ &+K_6(y-l_0/2) - K_7(y+3l_0/2) + K_8(y-3l_0/2) - K_9(y+3l_0/2) + K_{10}(y-3l_0/2)]\cdot\hat{\mathbf{a}}_z \end{aligned}.$$

(16)

For the second 11-segment loop antenna model:

$$\begin{cases} r_0 = \sqrt{x^2 + y^2 + z^2} = r \\ r_1 = \sqrt{(x-l_0)^2 + (y-l_0)^2 + z^2} = \sqrt{r^2 + 2l^2 - 2lr\sin\theta(\sin\phi + \cos\phi)} \\ r_2 = \sqrt{x^2 + (y-l_0)^2 + z^2} = \sqrt{r^2 + l^2 - 2lr\sin\theta\sin\phi} \\ r_3 = \sqrt{(x-l_0)^2 + (y+l_0)^2 + z^2} = \sqrt{r^2 + 2l^2 + 2lr\sin\theta(\sin\phi - \cos\phi)} \\ r_4 = \sqrt{(x+l_0)^2 + (y+l_0)^2 + z^2} = \sqrt{r^2 + 2l^2 + 2lr\sin\theta(\sin\phi + \cos\phi)} \\ r_5 = \sqrt{(x-\frac{3}{2}l_0)^2 + (y+\frac{1}{2}l_0)^2 + z^2} = \sqrt{r^2 + \frac{5}{2}l^2 + lr\sin\theta(\sin\phi - 3\cos\phi)} \\ r_6 = \sqrt{(x-\frac{3}{2}l_0)^2 + (y-\frac{1}{2}l_0)^2 + z^2} = \sqrt{r^2 + \frac{5}{2}l^2 - lr\sin\theta(\sin\phi + 3\cos\phi)} \\ r_7 = \sqrt{(x-\frac{1}{2}l_0)^2 + (y+\frac{3}{2}l_0)^2 + z^2} = \sqrt{r^2 + \frac{5}{2}l^2 + lr\sin\theta(3\sin\phi - \cos\phi)} \\ r_8 = \sqrt{(x-\frac{1}{2}l_0)^2 + (y-\frac{3}{2}l_0)^2 + z^2} = \sqrt{r^2 + \frac{5}{2}l^2 - lr\sin\theta(3\sin\phi + \cos\phi)} \\ r_9 = \sqrt{(x+\frac{1}{2}l_0)^2 + (y+\frac{3}{2}l_0)^2 + z^2} = \sqrt{r^2 + \frac{5}{2}l^2 + lr\sin\theta(3\sin\phi + \cos\phi)} \\ r_{10} = \sqrt{(x+\frac{1}{2}l_0)^2 + (y+\frac{1}{2}l_0)^2 + z^2} = \sqrt{r^2 + \frac{1}{2}l^2 + lr\sin\theta(\sin\phi + \cos\phi)} \end{cases}, \quad (17)$$

$$
\begin{cases}
K_0 = \dfrac{jkI_0 l_0 \cdot\ e^{-jkr}}{8\pi r^2} \\
K_1 = \dfrac{kI_0 l_0 e^{-jkr}}{8\pi r^2}(j - kl_0 \sin\theta(\sin\phi + \cos\phi)) \\
K_2 = \dfrac{kI_0 l_0 e^{-jkr}}{8\pi r^2}(j - kl_0 \sin\theta \sin\phi) \\
K_3 = \dfrac{kI_0 l_0 e^{-jkr}}{8\pi r^2}(j + kl_0 \sin\theta(\sin\phi - \cos\phi)) \\
K_4 = \dfrac{kI_0 l_0 e^{-jkr}}{8\pi r^2}(j + kl_0 \sin\theta(\sin\phi + \cos\phi)) \\
K_5 = \dfrac{kI_0 l_0 e^{-jkr}}{8\pi r^2}(j + \dfrac{1}{2} kl_0 \sin\theta(\sin\phi - 3\cos\phi)) \\
K_6 = \dfrac{kI_0 l_0 e^{-jkr}}{8\pi r^2}(j - \dfrac{1}{2} kl_0 \sin\theta(\sin\phi + 3\cos\phi)) \\
K_7 = \dfrac{kI_0 l_0 e^{-jkr}}{8\pi r^2}(j + \dfrac{1}{2} kl_0 \sin\theta(3\sin\phi - \cos\phi)) \\
K_8 = \dfrac{kI_0 l_0 e^{-jkr}}{8\pi r^2}(j - \dfrac{1}{2} kl_0 \sin\theta(3\sin\phi + \cos\phi)) \\
K_9 = \dfrac{kI_0 l_0 e^{-jkr}}{8\pi r^2}(j + \dfrac{1}{2} kl_0 \sin\theta(3\sin\phi + \cos\phi)) \\
K_{10} = \dfrac{kI_0 l_0 e^{-jkr}}{8\pi r^2}(j + \dfrac{1}{2} kl_0 \sin\theta(\sin\phi + \cos\phi))
\end{cases},
$$

(18)

$$
\begin{aligned}
\sum_{n=0}^{10} \mathbf{H}_n &= (K_0 - K_1 + K_2 - K_3 + K_4)\cdot z \cdot \hat{\mathbf{a}}_x + (K_5 - K_6 + K_7 - K_8 + K_9 - K_{10})\cdot z \cdot \hat{\mathbf{a}}_y \\
&+ [-K_0 x + K_1(x - l_0) - K_2 x + K_3(x - l_0) - K_4(x + l_0) - K_5(y + l_0/2) \\
&+ K_6(y - l_0/2) - K_7(y + 3l_0/2) + K_8(y - 3l_0/2) - K_9(y + 3l_0/2) + K_{10}(y + l_0/2)]\cdot \hat{\mathbf{a}}_z
\end{aligned}.
$$

(19)

Supplementary Note 4: Rectangular segment width calculation

The FPRFS is designed based on the conventional microstrip line structure to achieve arbitrary RF signal routing among different IO ports. It is critical to achieving a 50-ohm characteristic impedance for transmission line applications including IMNs. For an FR-4 substrate with a dielectric constant $\varepsilon_r = 4.28$ and a thickness of $h = 1.78$ mm, the effective dielectric constant is equal to[2]:

$$\varepsilon_e = \frac{\varepsilon_r + 1}{2} + \frac{\varepsilon_r - 1}{2} \frac{1}{\sqrt{1 + 12h/W}} = 3.269 \, . \quad \textbf{(20)}$$

The transmission line width $W = 3.5$ mm achieving $Z_0 = 50$ ohm is calculated from:

$$Z_0 = \frac{120\pi}{\sqrt{\varepsilon_e}[W/d + 1.393 + 0.667\ln(W/d + 1.444)]}, \quad W/d \geq 1. \quad \textbf{(21)}$$

Supplementary Note 5: Radian electrical length analysis for the IMN

The conversion of the transmission line's physical length into equivalent electrical length in radians is based on multiplying it with the wave number as follows, where effective dielectric constant should be taken into account as follows:

$$l_e = \beta l = k_0 \sqrt{\varepsilon_e} l = 2\pi \sqrt{\varepsilon_e} l / \lambda \, . \quad \textbf{(22)}$$

After being converted into radians, $\tan \beta l$ becomes periodic thus the repetitive transmission line segment pixel unit length should be carefully selected so that with different possible integer multiples of it, the corresponding wraparound radians are distributed evenly between 0 and π. This is the logic behind different segment unit lengths achieving different impedance matching capabilities. Therefore, an important principle for selecting the optimal l_{unit} for maximized load impedance being able to be matched is that the radian electrical lengths for all reconfigurable stub lengths are located relatively evenly between 0 and π. Based on the prototyped FPRFS, the resolution of the load-stub distance on the main line and any stub is five. Therefore, for the maximized load impedance area being able to be matched, it is ideal that all the possible radian electrical lengths l_n expressed below rounded between 0 and π are evenly distributed.

$$l_n = \beta \cdot \ l = \frac{2\pi \cdot \ \sqrt{\varepsilon_r} \cdot \ f}{c} \cdot \ l_{\text{unit}} \cdot \ n, \ n = 0,1,2,3,4,5 .$$

(23)

Some extreme examples are shown in Supplementary Fig. 4. At 2.45 GHz, when $l_{\text{unit}} = 17$ mm, all possible electrical lengths in radians are shown below:

$$l_n = \frac{2\pi \cdot \ \sqrt{\varepsilon_r} \cdot \ f}{c} \cdot \ l_{\text{unit}} \cdot \ n = \frac{2\pi \times \sqrt{3.2691} \times 2.45 \times 10^9}{3 \times 10^8} \times 0.017 \cdot \ n = \frac{1}{2}\pi \cdot \ n, \ n = 0,1,2,3,4,5 .$$

(24)

Thus, all load impedance circles overlap with the $k = 0.2$ concentric circle, as shown in Supplementary Fig. 4a, denoting that no load impedance other than the intrinsically matched impedance can be matched using the FPRFS even with great reconfigurability and abundant impedance matching network surface patterns.

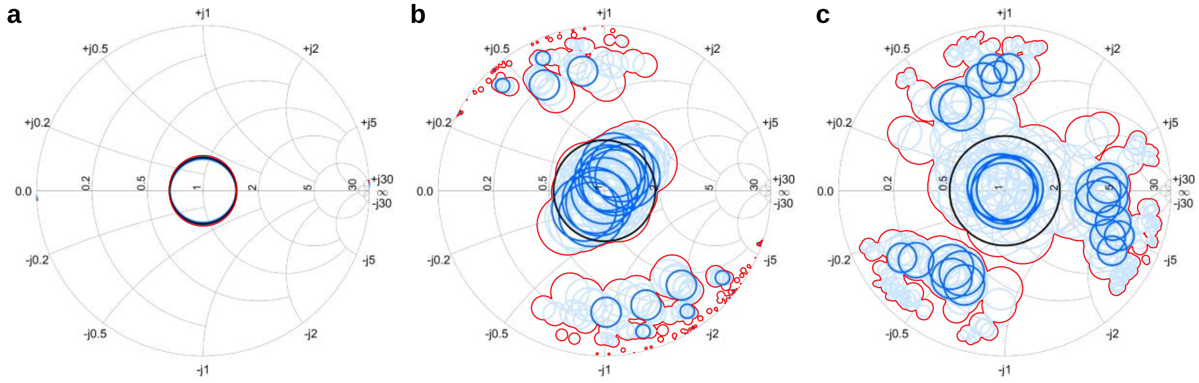

Supplementary Fig. 4 | Smith chart coverage with poorly selected transmission line segment unit length. a l_{unit} equals 17 mm where load impedance circles concentrating at Z_0, short circuit point, and open circuit point. **b** l_{unit} equals 50 mm where load impedance circles concentrating at Z_0 and only two main areas. **c** l_{unit} equals 45 mm where load impedance circles concentrating at Z_0 and only three main areas.

Similarly, when $l_{\text{unit}} = 50$ mm and $l_{\text{unit}} = 45$ mm, as can be seen from Supplementary Fig. 4b,c, the load impedance circles concentrate in two and three major areas respectively on the Smith chart. From the perspective of radian electrical lengths, when $l_{\text{unit}} = 50$ mm, l_n is quite close to 1.5π:

$$l_n = \frac{2\pi \cdot \sqrt{\varepsilon_r} \cdot f}{c} \cdot 0.05 \cdot n = 1.477\pi \cdot n \approx \frac{3}{2}\pi \cdot n, \; n = 0,1,2,3,4,5 \, .$$

(25)

When $l_{unit} = 45$ mm, l_n is quite close to 1.33π:

$$l_n = \frac{2\pi \cdot \sqrt{\varepsilon_r} \cdot f}{c} \cdot 0.045 \cdot n = 1.329\pi \cdot n \approx \frac{4}{3}\pi \cdot n, \; n = 0,1,2,3,4,5 \, .$$

(26)

The integer multiplies of these lengths will lead to repetitive radian electrical length thus causing increased redundancy with too many load impedance circles overlapping with each other. These kinds of transmission line segment unit length designs have poor impedance matching capability and are not capable of providing impedance matching for a wide range of loads.

Supplementary Note 6: Impedance matching capability calculation

Firstly, all IMN pattern corresponded inequalities are solved by Mathematica and the solutions are saved in text files. Secondly, MATLAB is used to parse the text-formatted solutions from Mathematica. In MATLAB, a blank Smith chart is plotted and converted into a bitmap file. The number of pixels inside the Smith chart is counted as the total area of the Smith chart. Thirdly, the parsed load impedance circles are plotted one by one (as shown in Supplementary Fig. 5a). After each plotting, the circle is converted into a bitmap, and the new bitmap is bitwise ANDed with the bitmap with all previously plotted circles to enlarge the circles-occupied area on the Smith chart.

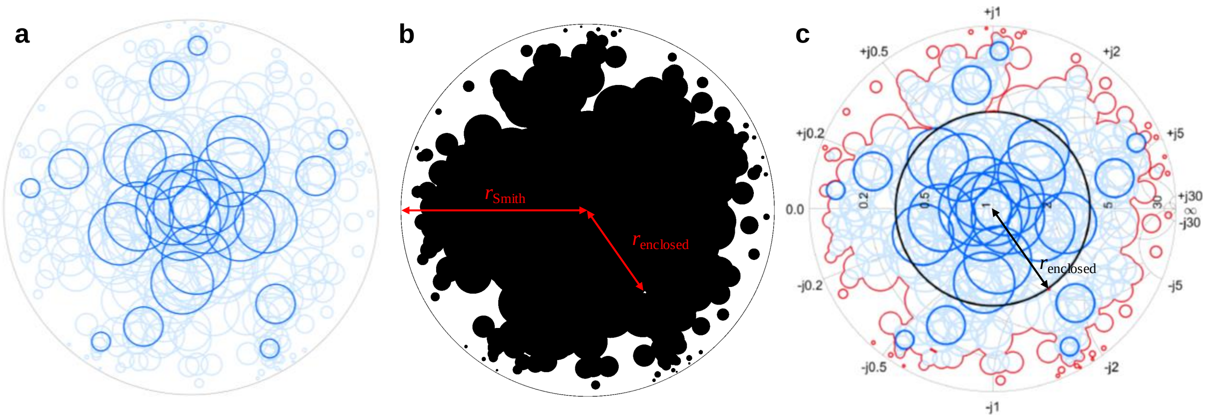

Supplementary Fig. 5 | Load impedance area calculation process. a Plotted load impedance circles (deep blue circles for single-stub matching cases and light blue circles for double -stub matching cases). **b** Converted load impedance bitmap for calculating occupied area percentage and largest enclosed concentric circle on the Smith chart. **c** Highlighting the outline in red showing the overall load impedance circles occupied areas on the Smith chart.

After going through this process, a bitmap plot as shown in Supplementary Fig. 5b is used for counting the total impedance circles-occupied area in pixels. Then the nearest unoccupied pixel is found to derive the radius of the largest enclosed concentric circle (as shown in the red circle in Supplementary Fig. 5c). Finally, the “bwboundaries” function in MATLAB is used to find and plot the outline.

Supplementary Note 7: Impedance matching bandwidth

The plotted circles can be used to analyze the theoretical impedance matching bandwidth for arbitrary loads. Dynamic bandwidth and static bandwidth are defined based on whether matching is achieved by constantly changing the FPRFS configuration or not. Given a specific load impedance shown on the Smith chart, if it is located inside one or more plotted circles, at least one FPRFS IMN pattern is capable of matching it into the acceptable matched region. Generally, for a load with reactance, its load impedance varies with frequency. On the Smith chart, it is plotted as a curve as frequency changes, as the black curve shown in Supplementary Fig. 6a,b for a dipole example. Defining VSWR being smaller than 1.5 as being properly matched then the dipole load is intrinsically matched between 2.24 GHz and 2.68 GHz. For each dot on the curve, find all circles enclosing it and plot them as shown in Supplementary Fig. 6a. The intersection of the load curve and the red contour outlining all these circles, excluding the grey dots' corresponding frequencies, is defined as the theoretical dynamic impedance matching bandwidth, denoting that at least one IMN pattern can be dynamically configured to match the load within it. In the example shown in

Supplementary Fig. 6a, the dynamic impedance matching bandwidth is about 6.6 times its intrinsic bandwidth. The segment pixel unit length of the FPRFS is designed to maximize its impedance matching capability, which also corresponds to wider dynamic impedance matching bandwidths for arbitrary loads.

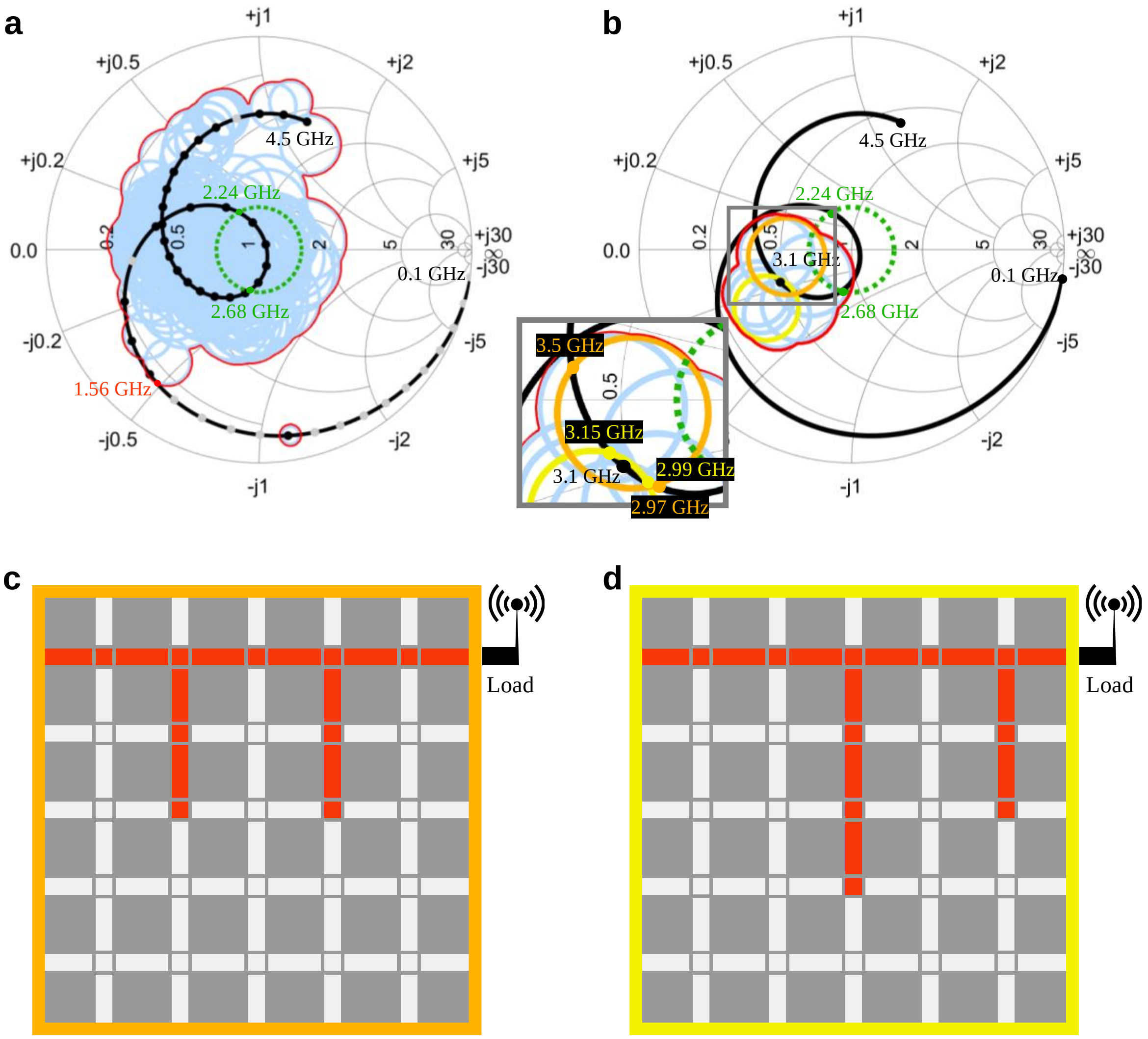

Supplementary Fig. 6 | FPRFS impedance matching static bandwidth analysis. a The Smith chart with a load dipole antenna curve (in black) and all impedance circles enclosing it from 100 MHz to 4.5 GHz with a 100 MHz step size. **b** All eight impedance circles enclosing the load dipole antenna impedance at 3.1 GHz; the inset shows the zoom-in graph of two highlighted impedance circles with different impedance matching static bandwidths. **c** The orange circle corresponded IMN pattern with wider static bandwidth. **d** The yellow circle corresponded IMN pattern with narrower static bandwidth.

For load dot enclosed by more than one circle, static impedance matching bandwidth is defined for each corresponding IMN pattern and compared horizontally. As the example shown in Supplementary Fig. 6b, there are altogether eight circles enclosing the load impedance at 3.1 GHz. The intersection of each circle and the load impedance curve is the static bandwidth for each corresponding IMN pattern. The orange and yellow circles in Supplementary Fig. 6b correspond to the IMN patterns shown in Supplementary Fig. 6c,d achieving the widest and narrowest static bandwidths. Although both IMN patterns manage to patch the load at 3.1 GHz, the orange IMN pattern implements a static impedance matching bandwidth of 0.53 GHz, which is about 3.3 times that of the yellow one.

Supplementary Note 8: FPRFS-based antenna's polarization programmability

The polarization programmability of the FPRFS-based antenna can be achieved through three approaches: modifying the antenna feeding position, changing the current distribution, and configuring multiple antenna elements excited with mutually related signals.

The positioning of the feeding point plays a crucial role in influencing the current distribution and electric field orientation across the antenna structure. This, in turn, dictates the characteristics of the electric field in the radiated electromagnetic wave, ultimately determining the antenna's orientation. As the example shown in Supplementary Fig. 7a, seven FPRFS emulated patch antennas with a length of 1 segment have different feeding positions. The simulation results in Supplementary Fig. 7b (column one) reveal far-field electric field vector trajectories above the

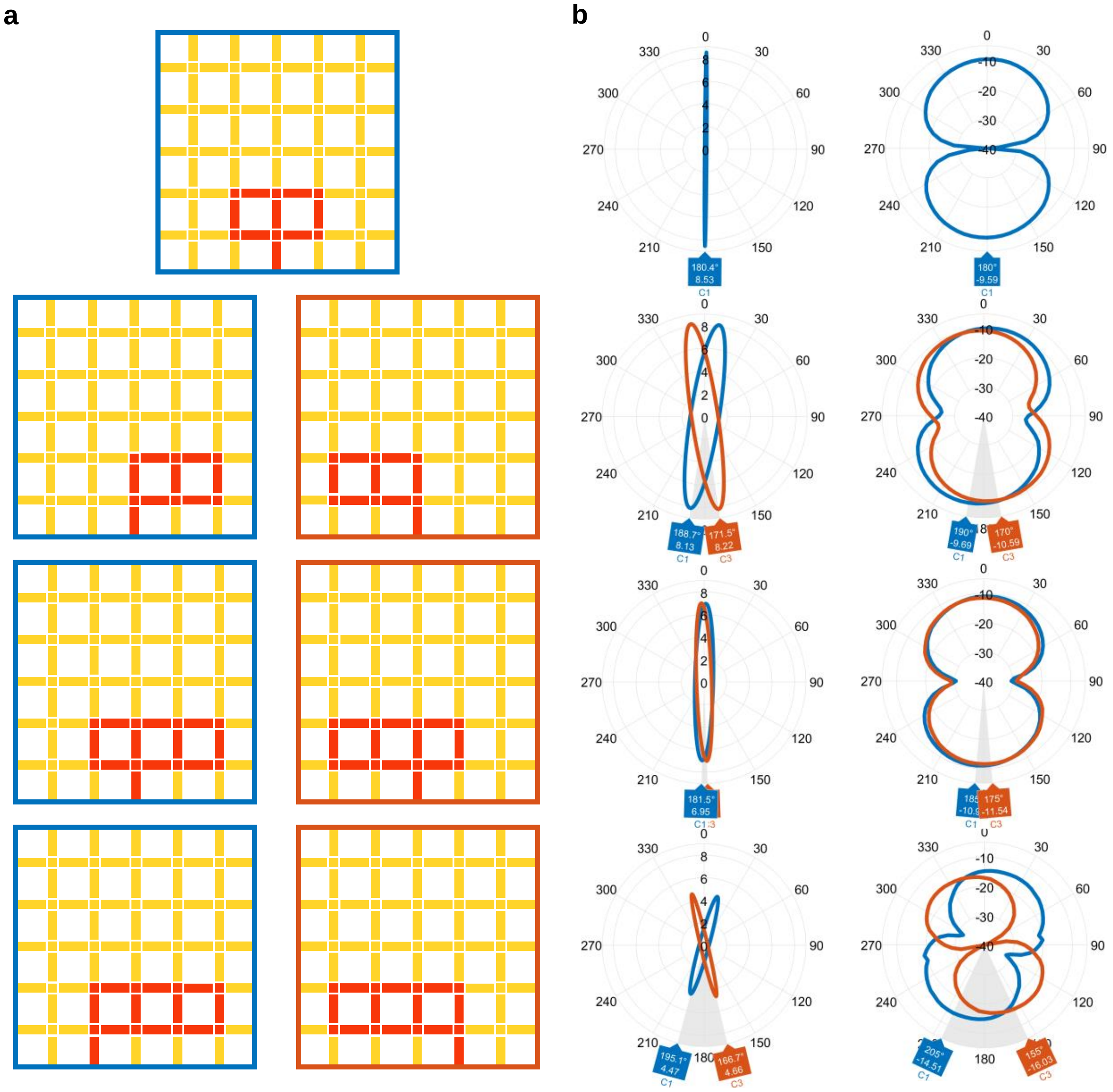

Supplementary Fig. 7 | FPRFS reconfigurable polarization based on varying feeding positions. a The FPRFS patch antenna patterns. **b** The simulated and measured polarization results for the corresponding FPRFS antenna patterns.

FPRFS at the TM_{10} mode frequency, showcasing two symmetric elliptical trajectories and a linear trajectory. The gain results measured by a rotating linear polarized antenna are included in Supplementary Fig. 7b (column two). Likewise, for other patch antenna patterns featuring multiple optional feeding positions, elliptical polarizations with varying axial ratios and orientations can be

dynamically reconfigured. These reconfigurations are supported by both simulated and measured results.

An FPRFS emulated fork antenna family is introduced to demonstrate the FPRFS antenna's polarization programmability in terms of modified current distribution. The fork antennas under test consist of a 1-segment long branch and a 2-segment long branch but with different orientations from the feeding point. The current distribution on the fork antenna structure is configurable by adjusting these orientations. The FPRFS fork antenna patterns shown in Supplementary Fig. 8a,b are simulated and measured at 2.45 GHz. Linear polarization and elliptical polarizations with different axial ratios and directions are configurable, as shown by the simulated and measured results in Supplementary Fig. 8c,d.

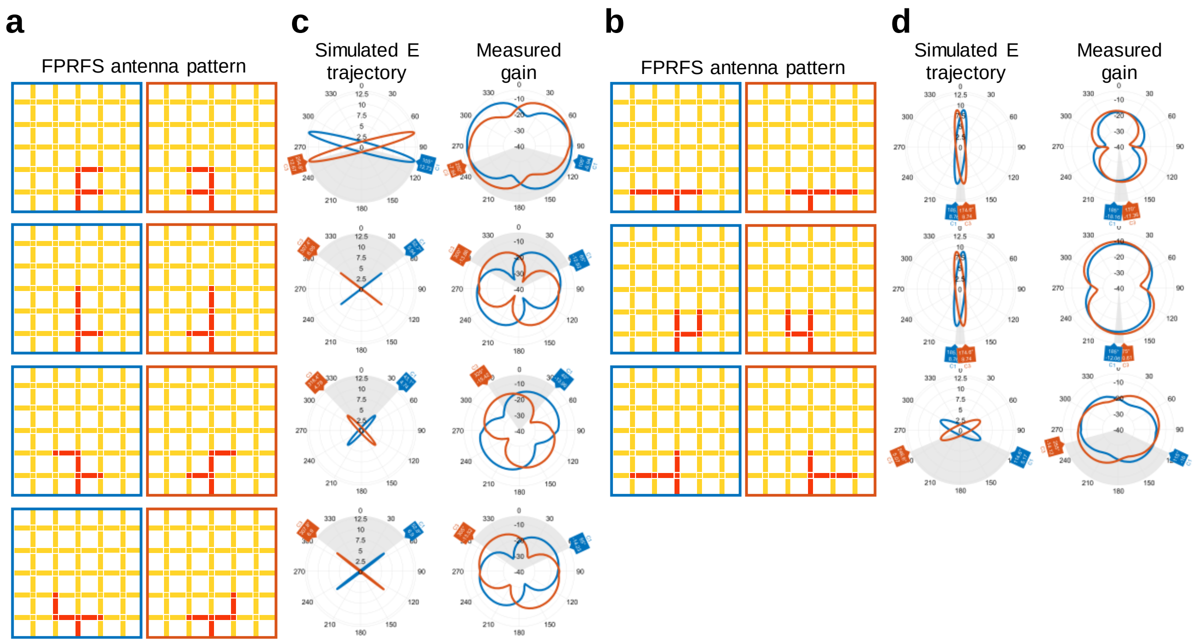

Supplementary Fig. 8 | FPRFS reconfigurable polarization based on varying current distributions. a,b FPRFS fork antenna patterns. **c,d** The simulated and measured polarization results for the corresponding FPRFS antenna patterns.

The third approach of programming multiple antenna elements resembles a phased array antenna. Multiple identical antenna elements can be configured on it, each with a distinct excitation port and source. The ability to tune phase delays to each element allows for dynamic polarization reconfiguration, enhancing the versatility and adaptability of the system. An example is shown in Supplementary Fig. 9, when two achiral 1×2 patch antenna elements are configured with separate feeding ports, the polarization is circular when the phase delay between these two excitation sources is orthogonal. When two chiral 1×2 patch antenna elements are configured and the delay

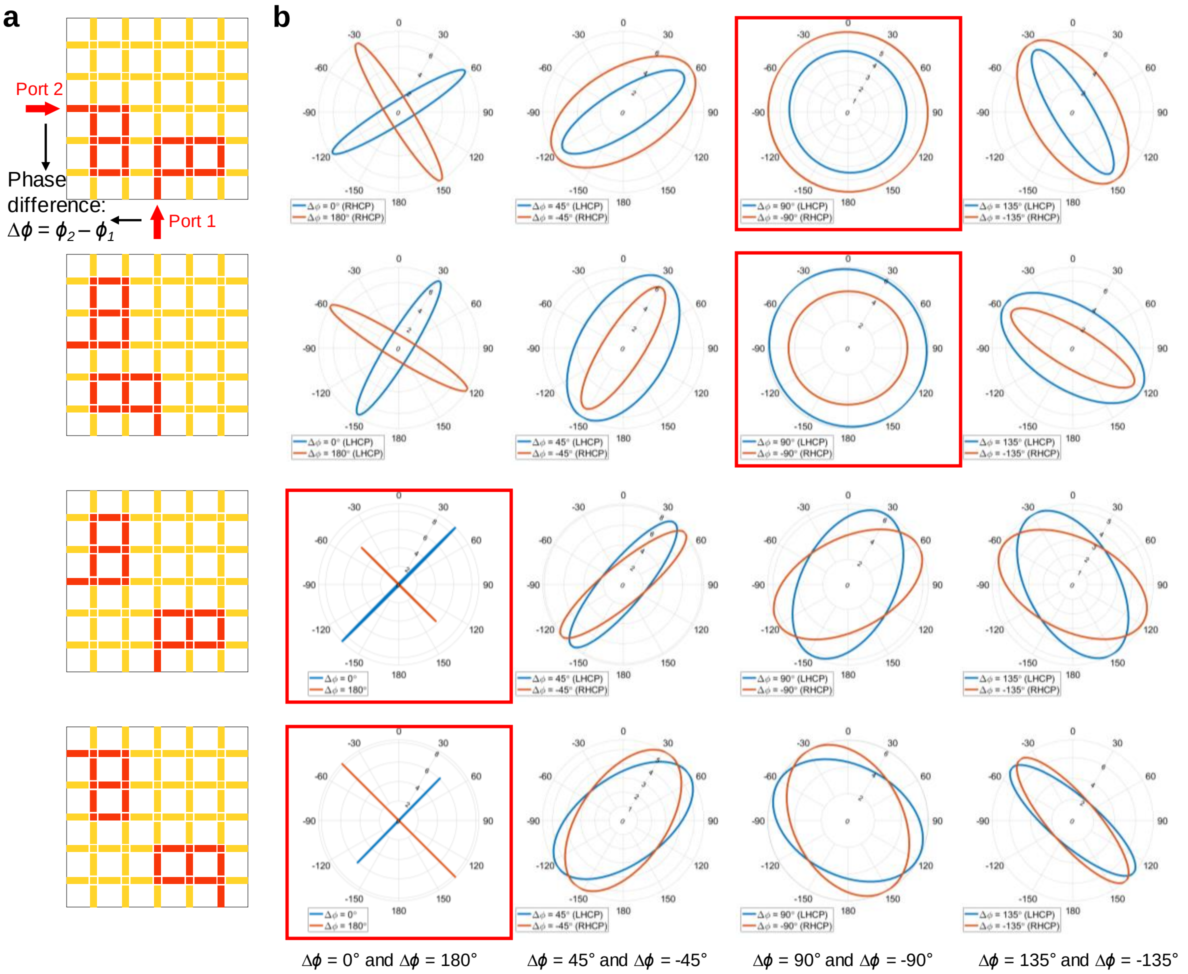

Supplementary Fig. 9 | FPRFS reconfigurable polarization based on feeding multiple antenna elements. a The FPRFS configuration of an antenna array with two identical 1×2 patch antenna elements. **b** The simulated polarization results of the FPRFS-based antenna array excited by two sources with identical amplitude but varying phase differences.

between them in-phase or out-of-phase, the polarization is linear. The polarization sense (LHCP or RHCP) can be configured by selecting either a leading or lagging phase difference between two excitations. Furthermore, the axial ratio and polarization orientation of elliptical polarizations can be tuned by adjusting the source phase differences, splitting power strength to each excitation port, and configuring the shape of individual antenna elements on the FPRFS.

Supplementary Note 9: PIN diode modeling and characterization

The PIN diode symbol and structure are shown in Supplementary Fig. 10a. The PIN diode lumped component models used for simulation are shown in Supplementary Fig. 10b[3,4].

Although PIN diodes are commonly used as RF switches in the gigahertz range, the switching behavior of PIN diodes is not strictly binary. When not biased or reverse biased, the PIN diode acts like a very low capacitance (smaller than 1 pF), which provides very high impedance for high

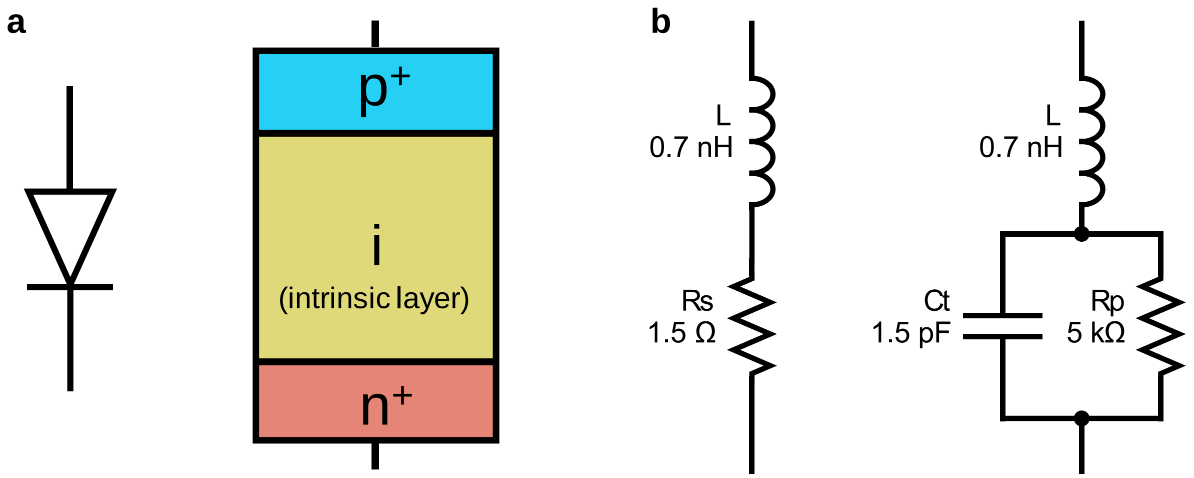

Supplementary Fig. 10 | PIN diode model. a The PIN diode symbol and semiconductor structure. **b** The lumped component model of the PIN diode in ON-state and OFF-state.

frequencies. When forward-biased, the PIN diode acts like a variable resistor, whose resistance has an almost linear relation with the biasing current. To verify the accuracy of the lumped component ON/OFF models of the PIN diode, a single-PIN diode and a double-PIN diode RF

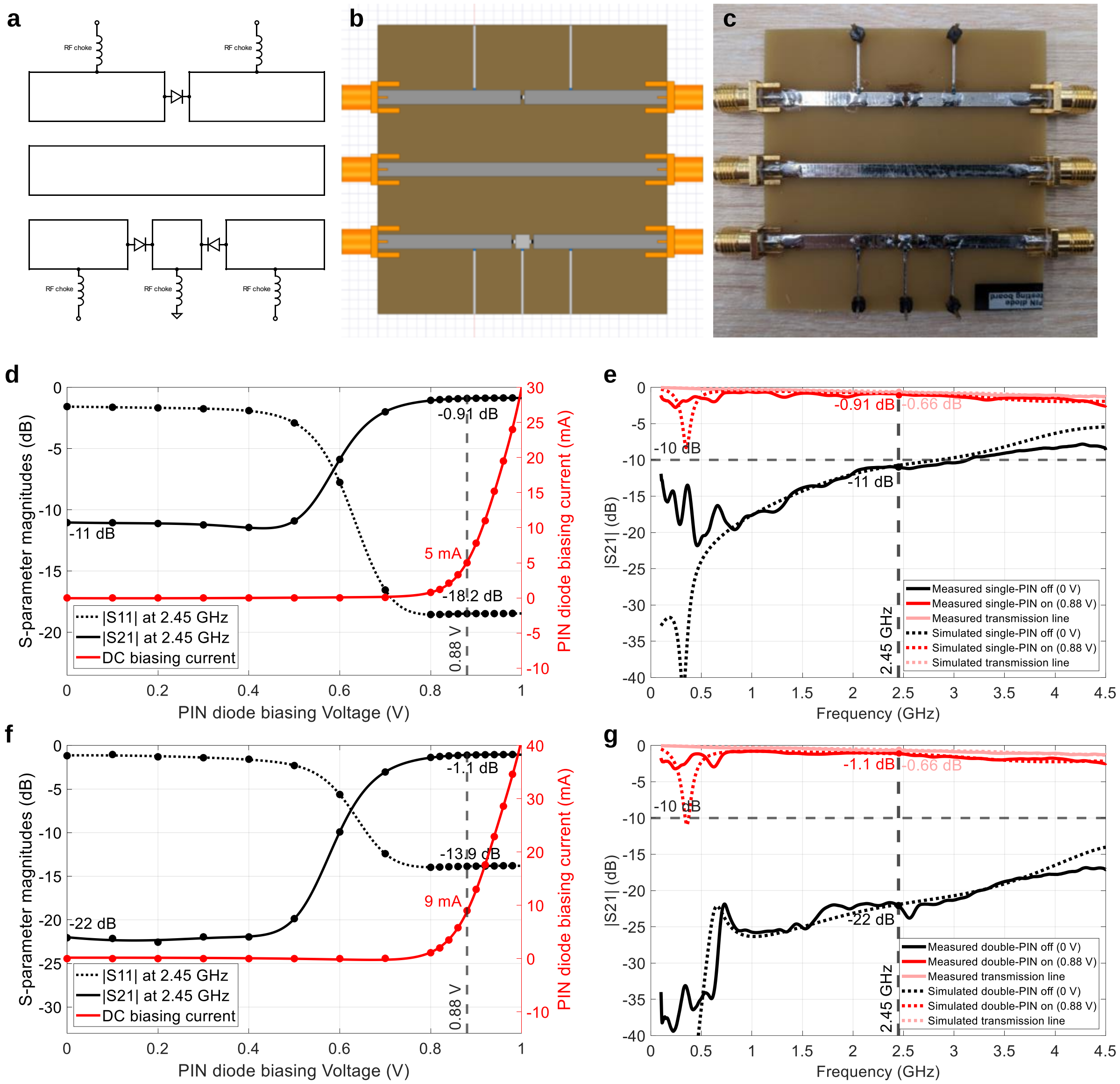

Supplementary Fig. 11 | PIN diode characterization results. a PIN diode-based RF switch circuit diagram. **b** PIN diode-based RF switch HFSS simulation model. **c** PIN diode-based RF switch testing circuit board. **d** The relationship between the single-PIN diode RF switch DC biasing voltage and the DC biasing current, and its effect on PIN diode insertion loss. **e** The simulated and measured ON/OFF behaviors of the single-PIN diode RF switch. **f** The relationship between the double-PIN diode RF switch DC biasing voltage and the DC biasing current, and its effect on PIN diode insertion loss. **g** The simulated and measured ON/OFF behaviors of the double-PIN diode RF switch.

switches are modeled in HFSS simulation and assembled in real life. The circuits for characterizing the PIN diode-based RF switches' behaviors are shown in Supplementary Fig. 11a-c. The relationship among the DC biasing voltage, DC bias current, and the insertion loss S21 for single-PIN diode and double-PIN diode RF switches are investigated and the results are plotted in Supplementary Fig. 11d,f. The measurements are taken with a 0.1 V interval from 0 V to 0.8 V PIN diode biasing voltage and with a 0.02 V interval from 0.8 V to 1 V PIN diode biasing voltage. It can be noticed that when the biasing voltage is below 0.5 V, the PIN diodes are fully OFF with an isolation S21 of below -10 dB for a single-PIN diode switch case and below -20 dB for a double-PIN switch case. The PIN diodes are fully ON when the biasing voltage is greater than 0.8 V. To keep the DC biasing power consumption as low as possible and also leave some safety margin for a decent ON-state insertion loss, the 0.8 V minimum turn-on voltage plus a small 0.08 V margin to contend with any voltage drops that may occur at any PIN diode, is finally selected (as the dashed line shown in Supplementary Fig. 11d,f) as the constant ON-state DC biasing voltage for all PIN diodes on the FPRFS in all experiments. At this biasing voltage, comparing the single-PIN diode switch and the double-PIN diode switch cases with the transmission line only without PIN diode switch case (the pink curve shown in Supplementary Fig. 11e,g), the introduced insertion loss per PIN diode in ON-state is about 0.2 dB at 2.45 GHz. However, a doubled extra isolation is achieved with the second PIN diode, as shown in Supplementary Fig. 11g. Thus, the double-PIN diode RF switch structure is applied for better compromised ON/OFF behaviors. The corresponding DC biasing current is thus about 4 to 5 mA per PIN diode. Checking the datasheet, the equivalent ON resistance with such a DC biasing current is $R_s = 1.5$ ohm. Under a 0 V DC biasing condition, the equivalent OFF resistance $R_p = 5000$ ohm is in parallel with a capacitor $C_T = 0.15$ pF as shown in Supplementary Fig. 10b. A parasitic packaging inductance of $L = 0.7$

nH is in series for both the ON and OFF models. This PIN diode lumped component model is used for all the simulations in this work and the average power consumption of an ON-state PIN diode is about 3 mW. As per the DC biasing IV curve, a minimum ON-state PIN diode power consumption of 0.53 mW (0.66 mA at 0.8 V) can be achieved with negligible insertion loss degradation.

The PIN diode characterization is also carried out with IP SMA connectors. The simulated and measured results are shown in Supplementary Fig. 12. The ON/OFF behavior of the PIN diode is not affected by the modified feeder configuration. Therefore, the lumped component model of PIN diodes still applies and the device power consumptions are the same. With the feeder polarity inverted, the results show an insertion loss increase of about 2 dB. This is attributed to the presence of a segmented ground plane over which the transmission line travels.

PIN diode characterization in NIP configuration is commonly used for testing, where the ON-state insertion loss and OFF-state isolation are determined. The standard NIP test's fundamental goal is to assess the power transmitted through the waveguide with the design aim of maximizing the

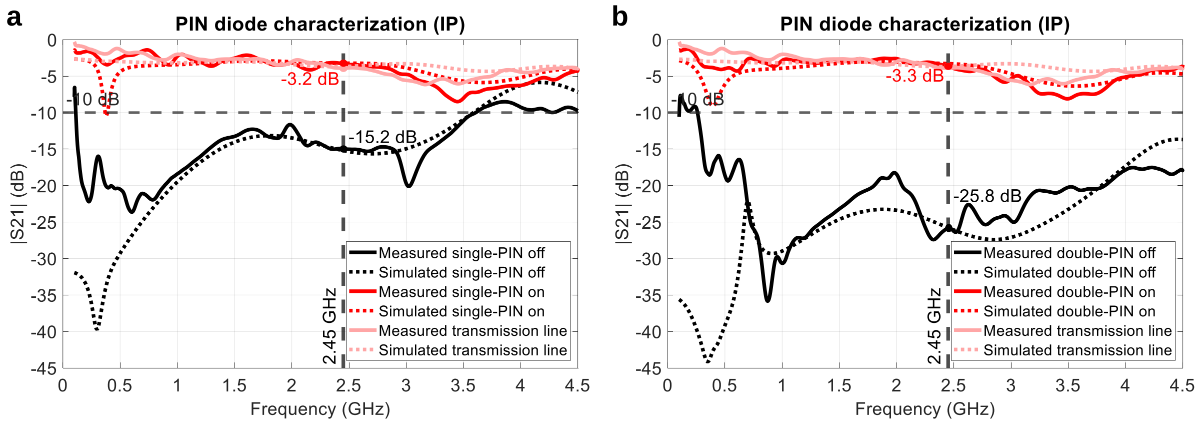

Supplementary Fig. 12 | PIN diode characterization results with IP feeders. a Simulated and measured ON/OFF behaviors of the single-PIN diode RF switch. **b** Simulated and measured ON/OFF behaviors of the double-PIN diode RF switch.

power transmitted in the on state whilst minimizing the power transmitted in the off state. A solid ground helps minimize power loss in the on state. It then follows that this application finds IP feeders with the segmented lossy ground undesirable. However, for antenna structures with PIN diodes, our goal is to increase radiation efficiency. Our improved antenna radiation performance is attributed to increased return current for the antenna application. Although the IP configuration makes the ground plane lossy, it also leads to increased return current and improved radiation performances. The relatively high OFF-state isolation is maintained, which is critical for confining the energy within the programmed conductive pattern cavity in IP cases, thus maintaining the IP FPRFS antenna's RF characteristic tunability.

Supplementary Note 10: The cruciform RF switching unit

As FPGAs use switching matrixes to flexibly route signals to different logic unit blocks, the FPRFS is composed of cruciform RF switching units for programmable routability for RF signals. The cruciform structure RF switching unit shown in Supplementary Fig. 13 enables a flexible

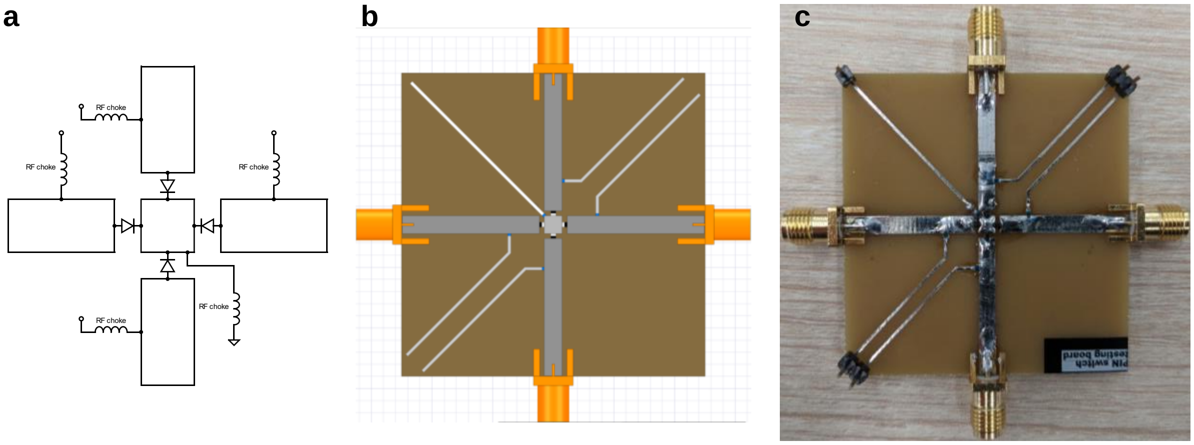

Supplementary Fig. 13 | The RF switching unit. a RF switching unit circuit diagram. **b** RF switching unit HFSS simulation model. **c** RF switching unit testing circuit board.

routing scheme of RF currents as shown in Supplementary Fig. 14. A scheme diagram of a single cruciform RF switching unit is shown in Supplementary Fig. 13a. A single cruciform RF switching unit circuit board as shown in Supplementary Fig. 13b,c is designed to demonstrate the RF signal routability and the PIN diode model accuracy. The four SMA ports are connected to a VNA in a

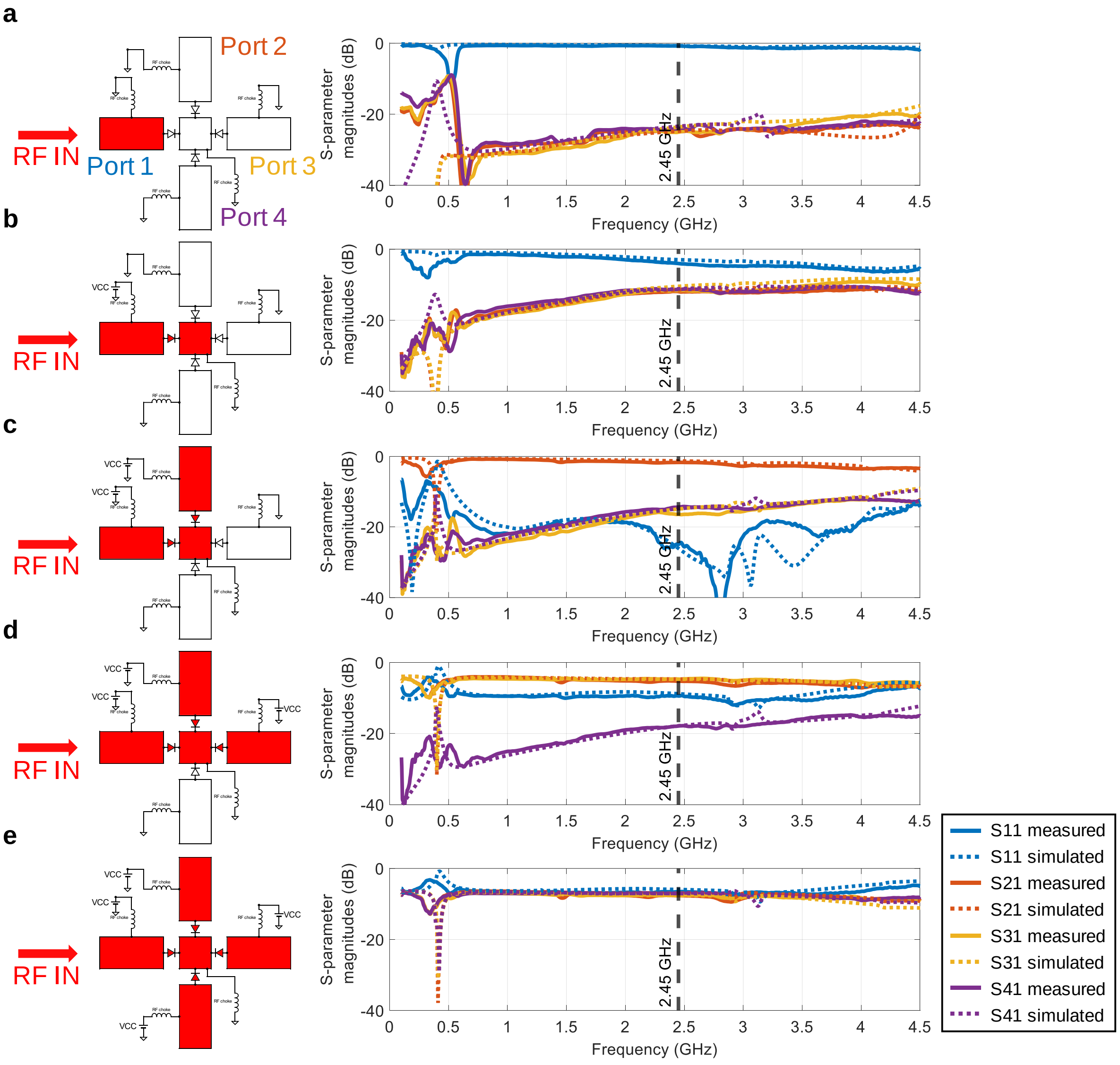

Supplementary Fig. 14 | RF switching unit characterization results. a Cruciform RF switching unit configured to be all OFF. **b** Cruciform RF switching unit configured as an open end with one segment being biased to VCC (red part denoting the active RF current path). **c** Cruciform RF switching unit configured as an RF current router with two segments being biased to VCC. **d** Cruciform RF switching unit configured as an RF current divider or combiner with three segments being biased to VCC. **e** Cruciform RF switching unit configured as an RF current divider or combiner with four segments being biased to VCC.

clockwise order starting with the port on the left and the RF power is only fed through SMA port 1. Therefore, by manipulating the ON/OFF behaviors of these four PIN diodes, the RF signal flow pattern as an open terminal (Supplementary Fig. 14a,b), as a signal router from one segment to another (Supplementary Fig. 14c), and as a signal divider (Supplementary Fig. 14d and

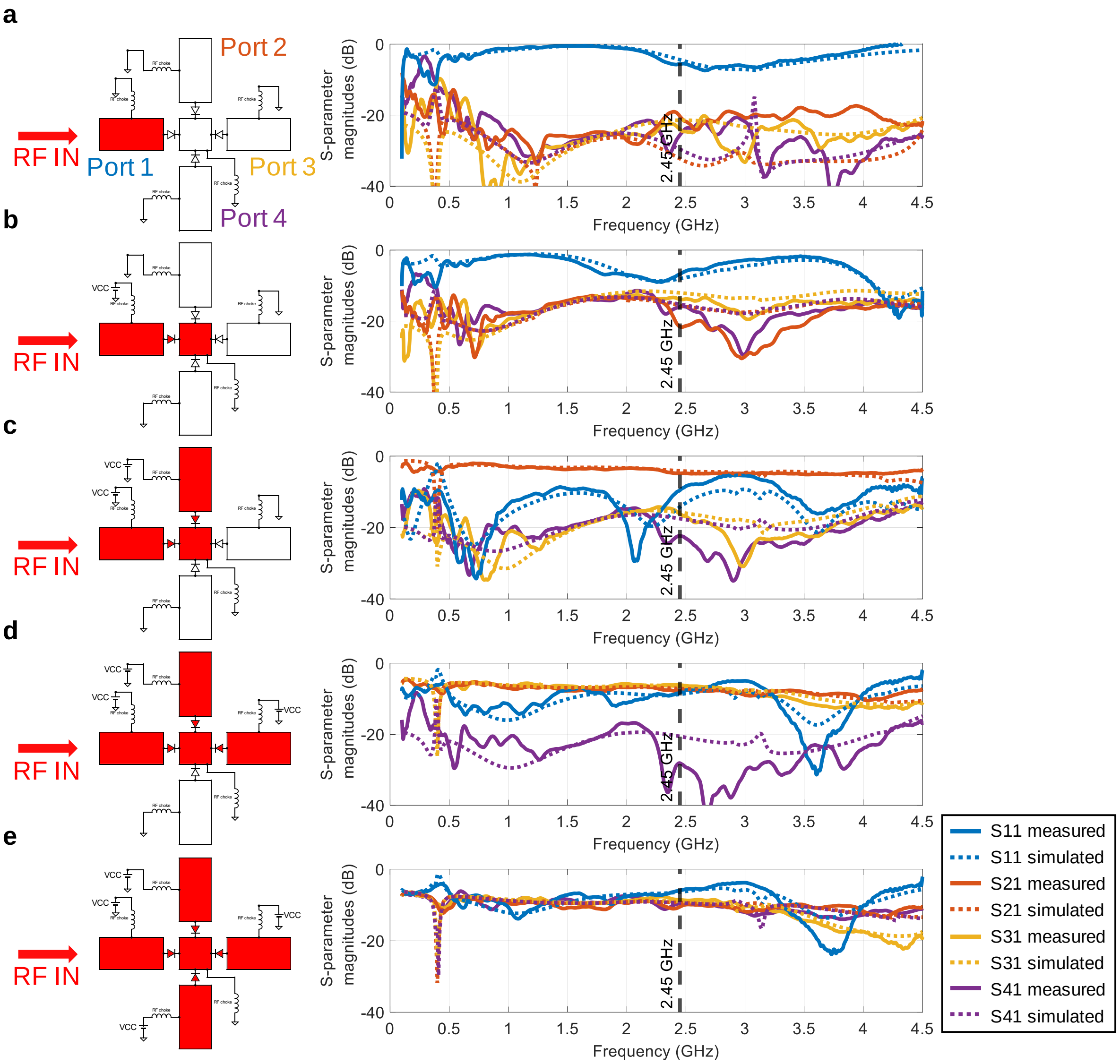

Supplementary Fig. 15 | RF switching unit characterization results with IP feeders. a Cruciform RF switching unit configured to be all OFF. **b** Cruciform RF switching unit configured as an open end with one segment being biased to VCC (red part denoting the active RF current path). **c** Cruciform RF switching unit configured as an RF current router with two segments being biased to VCC. **d** Cruciform RF switching unit configured as an RF current divider or combiner with three segments being biased to VCC. **e** Cruciform RF switching unit configured as an RF current divider or combiner with four segments being biased to VCC.

Supplementary Fig. 14e) can be achieved. The simulated and measured Scattering parameters are shown on the right. It is noticed that the insertion loss for a single PIN diode at 2.45 GHz is about 1 dB and for an RF signal to pass through a single cruciform RF switching unit, it must go through two PIN diodes. Compared with the double-PIN diode RF switch introduced in Supplementary Fig. 11, the insertion loss measured for the cruciform RF switching unit is higher (2 dB) because of the energy leakage caused by the other PIN diodes in the OFF state. This energy leakage through OFF-state PIN diodes contributes to the main power loss in the FPRFS structure. Similarly, in the frequency range below 700 MHz, both the simulated and measured Scattering parameter results show unexpected variations. This is because of the RF chokes for DC coupling. This effect is negligible since frequencies below 700 MHz are not in the FPRFS operating frequency range and the RF choke model will not be included in the simulations. All RF surface patterns are implemented by a combination of these basic cruciform RF switching unit patterns.

Similarly, the RF switching unit with IP connectors is also characterized and the results are shown in Supplementary Fig. 15. The signal routing capability is maintained with slightly higher insertion loss but better isolation.

Supplementary Note 11: Comparative analysis of antenna efficiency

The simulated and measured efficiency results for all 1×2-2-1 antennas (including REF, FPRFS with NIP and IP) are presented and discussed in the main text Fig. 5d. Supplementary Fig. 16a illustrates the measured peak gain as a function of frequency. Notably, the peak gain achieved by the IP FPRFS antenna exceeds that with NIP by more than 10 dBi and surpasses the conventional patch reference in most of the FPRFS dynamic bandwidth.

Similar improved radiation performance trends are observed for patch antenna patterns with different lengths (2×2-2-1 and 3×2-2-1) in Supplementary Fig. 16b,c, emphasizing the benefits of

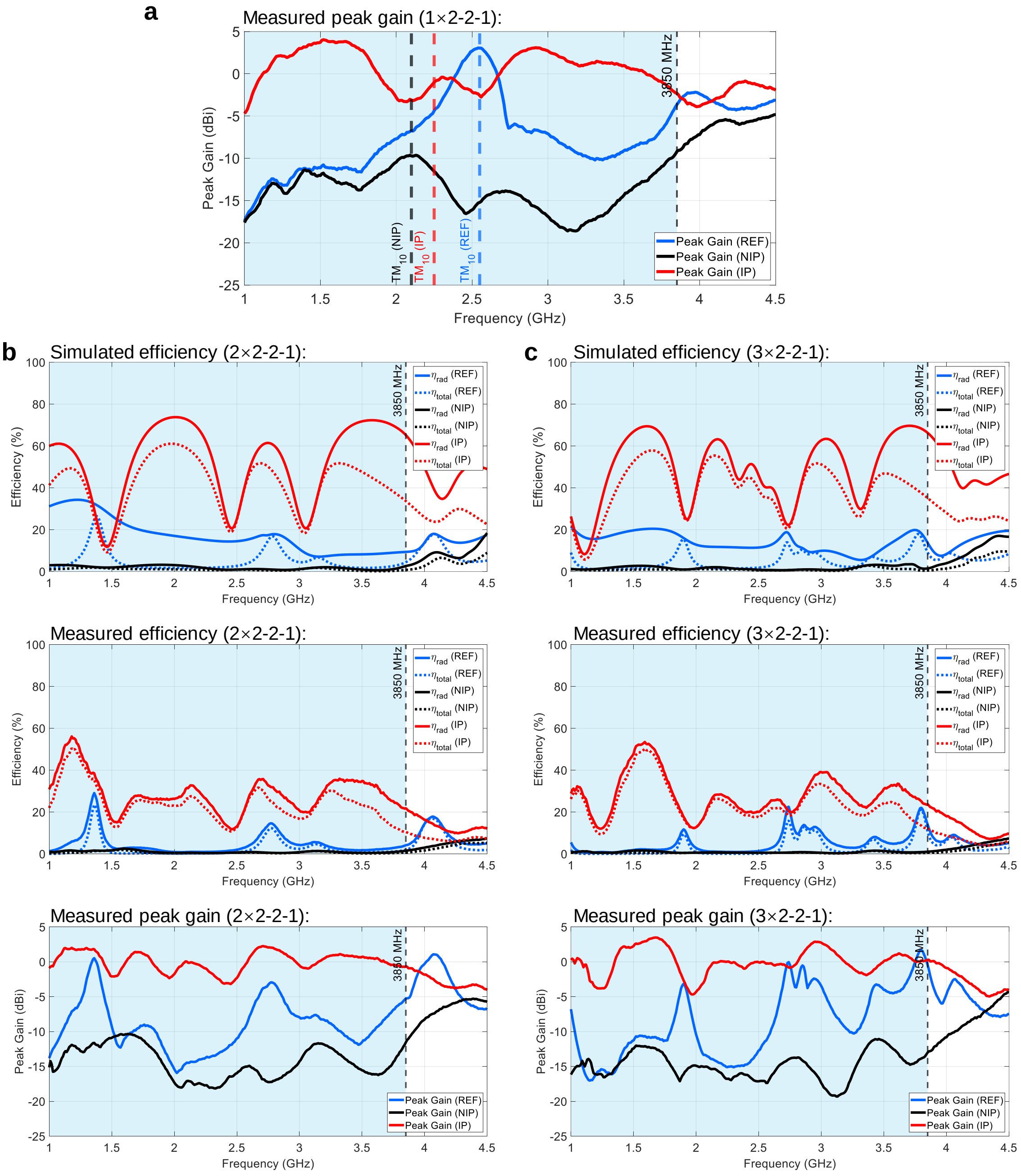

Supplementary Fig. 16 | FPRFS antenna efficiency results. a Measured peak gain as a function of operating frequency for FPRFS emulated and conventional 1×2-2-1 patch. **b** Simulated efficiency, measured efficiency, and measured peak gain as a function of operating frequency for FPRFS emulated and conventional 2×2-2-1 patch. **c** Simulated efficiency, measured efficiency, and measured peak gain as a function of operating frequency for FPRFS emulated and conventional 3×2-2-1 patch.

IP. A comparison with simulated efficiency results reveals identical curve trends with slightly lower values in the measured efficiency results. The discrepancy arises from variations in the length of the coaxial feeding cable between experiments and simulations.

In summary, both the efficiency and peak gain results of the FPRFS antenna with IP outperform those of the FPRFS antenna with NIP and its reference antenna counterparts across the entire FPRFS operating spectrum. Conventional patches only exhibit comparable results with the FPRFS IP antenna in their TM mode operating frequencies. These findings are further refined and visualized as histograms in the main text Fig. 5e-j.

Supplementary Note 12: Reconfigurable antenna efficiency comparison

The efficiencies achieved by reconfigurable antennas vary depending on their substrate, operating frequency, switching mechanism, RF switch characteristics, and reconfigurability of the antenna structure. Below is a comparison table.

Supplementary Table. 1 | Reconfigurable antenna efficiency comparison

Literature	Switching mechanism	Number of RF switches	Radiation efficiency	Other aspects
Feng et al.(2020)[3]	Reflective metasurface	NA	41%-51%	Require other RF sources to function.
Jin et al.(2018)[4]	PIN diode	4	<60%	Bandwidth: 2.25-3.16 GHz<br>Total number of states: 4
Abutarboush et al.(2018)[5]	PIN diode	2	40%-50%	Bandwidth: 1.5-4 GHz<br>Total number of states: 4
Sheta et al.(2008)[6]	PIN diode	3	20%-58%	Bandwidth: 620-1150 MHz<br>Total number of states: 4
Hussain et al.(2015)[7]	PIN diode	4	22%-40%	Bandwidth: 710-3600 MHz<br>Total number of states: 4
This work	PIN diode	100	22%-43% (IP);<br>1.2%-1.8% (NIP)	Bandwidth: 850-3850 MHz<br>Total number of states: 2^{60}

The reflective metasurface presented by Feng et al. is a special type of reconfigurable RF devices, which requires other RF sources to function. Except for reconfigurable metasurfaces, in the other published work, their antenna reconfigurabilities are very limited. We conjecture that the major reason for this is the escalation of loss associated with the growing number of RF switches. The limitation imposed by an increasing number of RF switches on compromised radiation efficiency is overcome in our work, unlocking significantly increased potential in flexibility, programmability, and scalability for reconfigurable antennas. Others' work achieved comparatively high radiation efficiency but their antenna reconfigurabilities are constrained by very few lossy RF switches. Although the current radiation efficiency achieved in our work is comparable to state-of-the-art reconfigurable antennas, an important point is that our efficiency does not degrade as the number of RF switches increases. Our efficiency can be further increased by replacing the current FR4 substrate with a Rogers substrate with a lower dielectric constant and tangent loss and making the substrate thinner.

Supplementary Note 13: Integrating Advanced Optimization Algorithms

Optimizing pixelated surfaces like our proposed FPRFS involves a binary multi-dimensional problem[5]. Artificial intelligence (AI) has been explored in various application areas for adaptive reconfigurable antenna arrays, including adaptive nulling, wireless localization, multiple-input multiple-output (MIMO) communications, element failures, and calibration[6]. The configuration of our pixelated FPRFS is solely determined by the ON/OFF patterns of all PIN diodes. Therefore, a single-bit binary array representing the ON/OFF states of all PIN diodes can serve as the optimization output (with '1' representing ON and '0' representing OFF). A neural network model can be developed to take desired RF characteristics, such as VSWR and radiation pattern, as input,

and the optimized configuration as output. This model can be trained using a dataset containing FPRFS configurations and their corresponding RF characteristics.

Similar applications of optimizing reconfigurable RF devices using advanced optimization techniques have been widely explored with satisfactory results. Alkurt et al. (2021) presented a reconfigurable ground plane for a monopole antenna that can achieve the desired 3D antenna with the help of an ANN[7]. The reconfigurable ground plane consists of an 18×18 matrix of unit cells and each cell is represented by logic 1 or 0 for its states. The ANN is trained by 152 different configurations with simulated results, and takes S11 level, maximum radiation direction and gain as input parameters to generate the ground plane configuration as output. Montaser et al. (2022) built a Neural Network-based that can learn and predict the far-field radiation pattern of a designed metasurface with an array of reconfigurable unit cells[8]. This method predicts the radiation pattern with high accuracy in a substantially shorter time period and can be integrated with adaptive reconfigurable RF designs effectively. Noh et al. (2022) presented a deep neural network (DNN) that can predict the radiation pattern of a reconfigurable metasurface and search for the best unit cell configuration for beam forming[9]. This DNN-based approach is highly efficiency in managing massive search volumes and is advantageous for complex active unit arrays like reconfigurable antennas. Kovaleva et al. (2020) presented a cross-entropy method to optimize the configuration of a metasurface for artificial magnetic conductor and phase shifter applications with desired characteristics[5].

Supplementary Note 14: Direction coupler modeling and characterization

The directional coupler as shown in Supplementary Fig. 17a is modeled with insertion loss $\mathrm{IL}(f)$, coupling $\mathrm{CP}(f)$, and isolation $\mathrm{IS}(f)$. All these can be directly measured by a VNA and the results are shown in Supplementary Fig. 17b. The insertion loss and coupling of the directional coupler are very consistent across the entire frequency spectrum under the test. The isolation is generally lower than -30 dB and increases with frequency. These characterized directional coupler model information are saved in the format of $R + jX$ in advance.

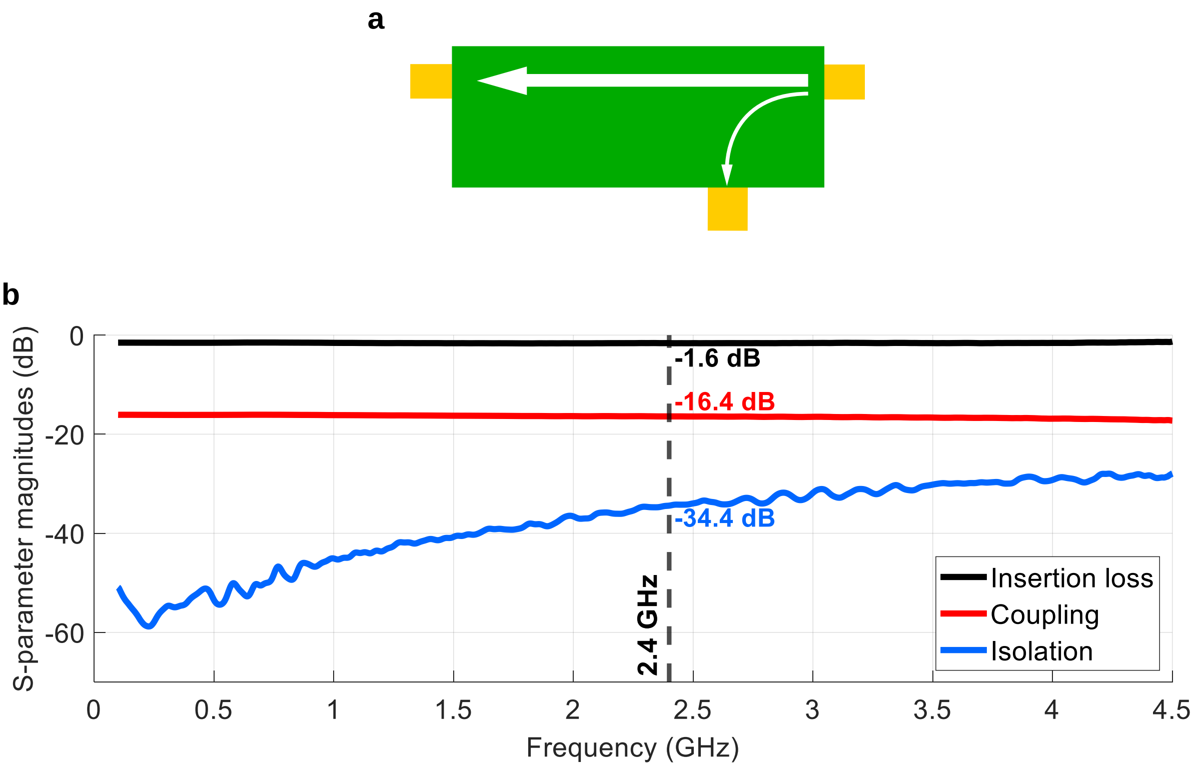

Supplementary Fig. 17 | Directional coupler characterization results. a Topology of the directional coupler used in the experimental setup. **b** S-parameter characterization for the 3-port directional coupler.

Supplementary Note 15: Experimental setup discussion

Two experimental setups are applied for the self-optimization and self-adaptation tests: the RF meter-based setup (shown in Supplementary Fig. 18a,b,d) and the VNA-based setup (shown in Supplementary Fig. 18c). The VNA-based self-optimization setup as shown in the main text Fig. 7d and Supplementary Fig. 18c is more convenient for experimental testing purposes as the VNA's spectrum covers a range of frequencies and the self-optimization for all frequencies inside the spectrum can be carried out and saved by a single run. The drawback is that the response time for the system to return the optimized FPRFS surface pattern for any frequency is slower also because of the VNA's spectrum sweeping mechanism and the serial communication between the VNA and the PC for large volumes of data. The RF meter-based setup is closer to the practical self-optimization and self-adaptive scenarios. In this setup as shown in Supplementary Fig. 18a,b, an RF signal generator is employed to simulate an incident power, replicating the role of a transmitter in providing input signals to the antenna. The RF meter, depending on where it is located (the TX side, as shown in Supplementary Fig. 18d, or the RX side, as shown in Supplementary Fig. 18b), measures the locally reflected power or the far-field power radiation strength, which is used as feedback for iterating the optimal FPRFS configuration under a certain circumstance. For instance, for finding the optimal FPRFS IMN surface patterns at three distinct frequencies as shown in Fig. 7g, using the VNA-based setup finds all three configurations within one single FPRFS pattern sweeping cycle and the solutions are saved and can be recalled accordingly. For the RF meter-based setup as shown in Fig.8b, the signal generator as the source needs to be set to 1.2 GHz, 1.8 GHz, and 2.4 GHz and run three FPRFS pattern sweeping cycles respectively. The RF meter-based

setup suits better in practical scenarios with sources featuring complex frequency components and requiring a fast response.

Both setups are introduced and experimentally demonstrated in this work. They are not utilized simultaneously within a single setup but are instead applied in different cases. The VNA-based setup primarily serves experimental testing purposes, while the RF meter-based setup represents practical application scenarios. Notably, the VNA-based setup exhibits a slower response compared to the latter.

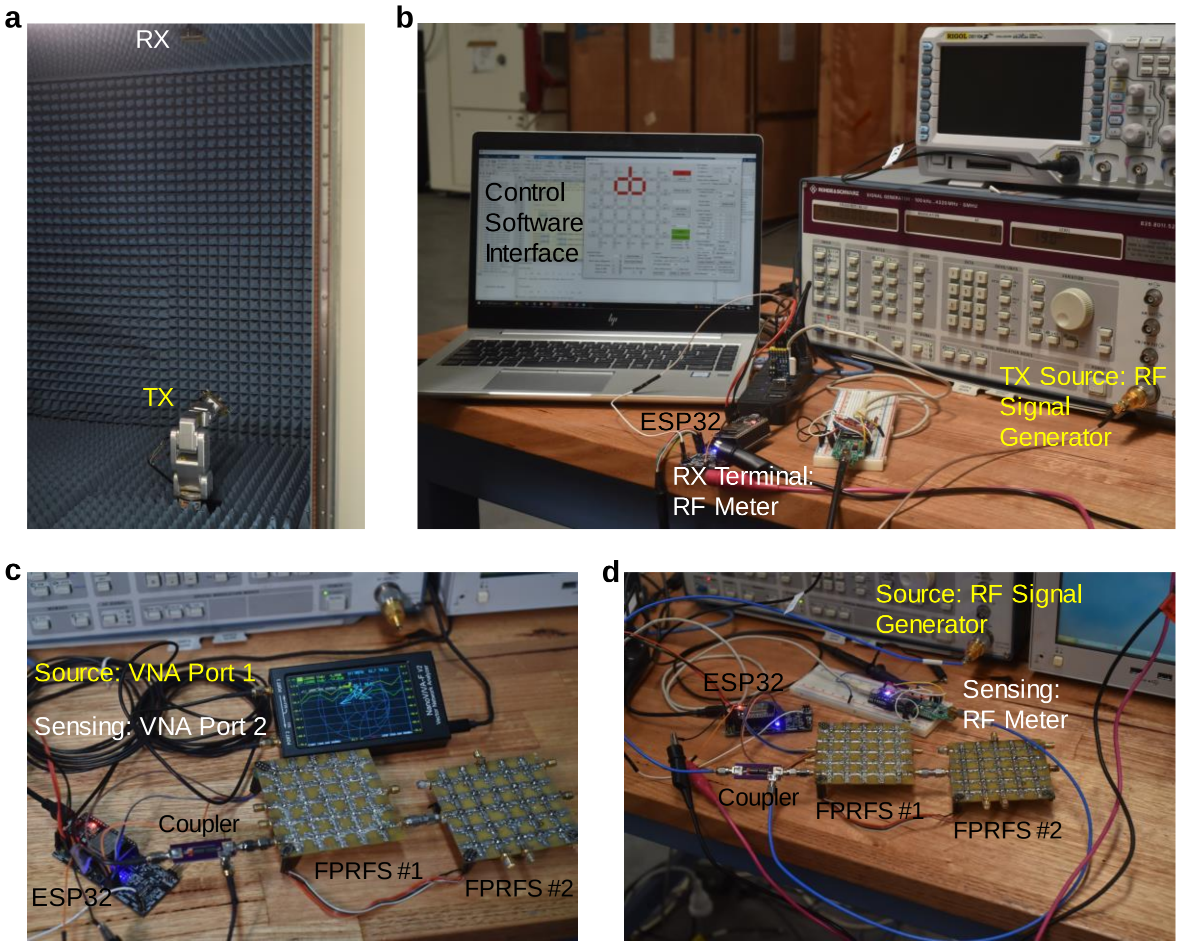

Supplementary Fig. 18 | Experimental setups. a,**b** Experimental setup for self-optimizing FPRFS antenna (TX) gain and radiation pattern at the target location (RX). **c** Experimental setup 1 for self-optimizing VSWR using a VNA. **d** Experimental setup 2 for self-optimizing VSWR using a signal generator and an RF meter.

In terms of practicality, the VNA-based setup is irrelevant and the signal strength-based sensing scheme is applied for both the local VSWR optimization and far-field radiation characteristics optimization. As depicted in Fig. 1b, assume that a part of the FPRFS is programmed to do impedance matching and a part to work as an antenna. When the self-optimization process starts, on the VSWR optimization side, the aim is to minimize the locally coupled reflected power thus maximizing the incident power to the FPRFS antenna part. On the radiation gain optimization side, the aim is to maximize the SNR at the target base station, which relies on the channel estimation process generating the CSI and sending it back to the transmitter. These two self-optimization processes are closely interrelated and mutually influential and are ideally carried out simultaneously with a judicious algorithm, dynamic decision-making mechanism, and iterative process.

Supplementary Note 16: Integrating multi-objective optimization methods

In the realm of reconfigurable antennas, achieving optimal performance requires a delicate balance across multiple RF characteristics, including radiation gain, efficiency, directivity, VSWR, and signal-to-noise ratio (SNR), among others. Traditionally, the optimization process focused on individual parameters, often resulting in suboptimal solutions that failed to address the intricate interplay between these diverse factors. As the development of reconfigurable RF devices with increasing flexibility like our FPRFS, there arises a pressing need for an approach to comprehensively optimize multiple antenna characteristic parameters. This necessity has paved the way for the adoption of multi-objective optimization (MOO) methods, which enable the simultaneous consideration of multiple conflicting objectives. MOO problems are commonly faced in reconfigurable RF applications due to the large number of tuning parameters. Zapata Cano

et al. (2023) presented an enhanced MOO algorithm offering a flexible design approach for electromechanically adaptable devices leveraging pixelated structures[10].

Our current solution is to assign different weights to different parameters, which belong to the scaled multi-objective optimization (MOO) method[11]. This method has three types of weights: equal weights, rank-order centroid weights, and rank-sum weights. In our case, different weights are assigned depending on their priorities to achieve the optimal combination of SNR at receivers and VSWR at the transmitter. Different weights are assigned and sent to different receiving base stations to achieve a radiation pattern with the optimal SNRs at multiple base stations. Different weights are assigned to different target frequencies to shape the comprehensive VSWR curve at multiple frequencies of interest.

Supplementary Note 17: FPRFS control and visualization software interface

The complementary FPRFS control software interface is created based on the MATLAB GUI. The interface consists of six panels and their functions are shown in Supplementary Fig. 19.

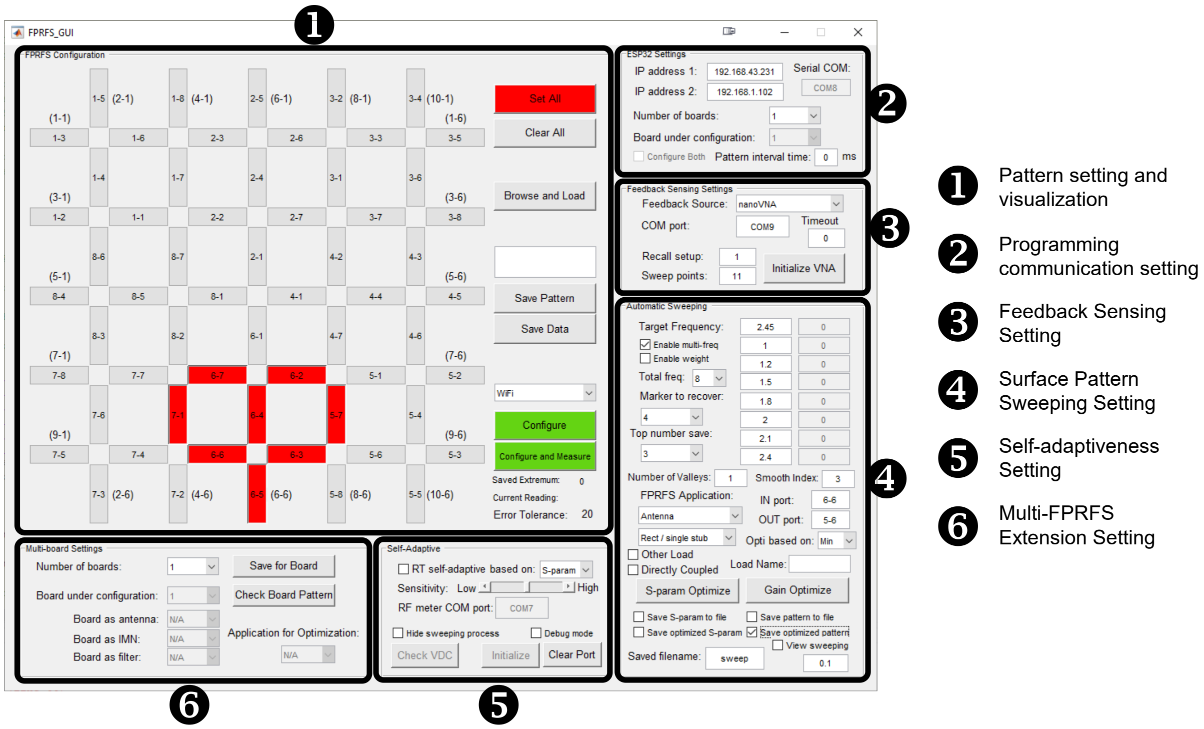

Supplementary Fig. 19 | The control and visualization software GUI for the FPRFS.

Supplementary References

1. Balanis, C. A. Antenna Theory: A Review. *Proc. IEEE* **80**, 7–23 (1992).

2. Pozar, D. M. *Microwave engineering*. (Fourth edition. Hoboken, NJ : Wiley, [2012] ©2012).

3. Lim, I. & Lim, S. Monopole-Like and boresight pattern reconfigurable antenna. *IEEE Trans. Antennas Propag.* **61**, 5854–5859 (2013).

4. Iqbal, A. *et al.* Frequency and pattern reconfigurable antenna for emerging wireless communication systems. *Electron.* **8**, 3–14 (2019).

5. Kovaleva, M., Bulger, D. & Esselle, K. P. Cross-Entropy Method for Design and Optimization of Pixelated Metasurfaces. *IEEE Access* **8**, 224922–224931 (2020).

6. Zardi, F., Nayeri, P., Rocca, P. & Haupt, R. Artificial Intelligence for Adaptive and Reconfigurable Antenna Arrays: A Review. *IEEE Antennas Propag. Mag.* **63**, 28–38 (2021).

7. Alkurt, F. O., Erkinay Ozdemir, M., Akgol, O. & Karaaslan, M. Ground plane design configuration estimation of 4.9 GHz reconfigurable monopole antenna for desired radiation features using artificial neural network. *Int. J. RF Microw. Comput. Eng.* **31**, 1–12 (2021).

8. Montaser, A. M. & Mahmoud, K. R. Design of Intelligence Reflector Metasurface Using Deep Learning Neural Network for 6G Adaptive Beamforming. *IEEE Access* **10**, 117900–117913 (2022).

9. Noh, J. *et al.* Reconfigurable reflective metasurface reinforced by optimizing mutual

coupling based on a deep neural network. *Photonics Nanostructures - Fundam. Appl.* **52**, (2022).

10. Zapata Cano, P. H. *et al.* Ultra-Low-loss Reconfigurable Phase-shifting Metasurface in V band: A Multi-objective Optimization Approach. *IEEE Trans. Antennas Propag.* **14**, 1–1 (2023).

11. Gunantara, N. A review of multi-objective optimization: Methods and its applications. *Cogent Eng.* **5**, 1–16 (2018).
