# Supplementary material for: A multifunctional smart field-programmable radio frequency surface
Source: Nat Commun. 2024 May 13;15:4042. doi: 10.1038/s41467-024-48242-z (PMC11091106; doi:10.1038/s41467-024-48242-z)
Supplement: Supplementary file 3 — Description of Additional Supplementary Files [file 41467_2024_48242_MOESM3_ESM.pdf]

Description of Additional Supplementary Files

Supplementary Movie 1: FPRFS patch antenna pattern sweeping process.

Supplementary Movie 2: FPRFS impedance matching network pattern sweeping process.

Supplementary Movie 3: Matching an FPRFS antenna load at 2.4 GHz with a single stub using a nanoVNA setup.

Supplementary Movie 4: Matching an FPRFS antenna load at 2.4 GHz with a single stub using an RF meter setup.

Supplementary Movie 5: Self-adaptation of the FPRFS antenna against far-field variations.

Supplementary Movie 6: Self-adaptation of the FPRFS antenna against near-field loading effects.

Supplementary Movie 7: Self-adaptation of the FPRFS antenna against structural damages.

Supplementary Movie 8: Self-adaptation of the FPRFS antenna to the presence of water.

Supplementary Movie 9: Self-adaptation of the FPRFS impedance matching network to the presence of water on the load.

Supplementary Movie 10: Self-adaptation of the FPRFS impedance matching network to the presence of water on itself.
